# TFAM expression in brown adipocytes confers obesity resistance by secreting extracellular vesicles that promote self-activation

**DOI:** 10.1016/j.isci.2022.104889

**Published:** 2022-08-10

**Authors:** Masakazu Fujii, Daiki Setoyama, Kazuhito Gotoh, Yushi Dozono, Mikako Yagi, Masataka Ikeda, Tomomi Ide, Takeshi Uchiumi, Dongchon Kang

**Affiliations:** 1 Department of Clinical Chemistry and Laboratory Medicine, Graduate School of Medical Sciences, Kyushu University, Fukuoka 812-8582, Japan; 2 Department of Internal Medicine, Fukuoka Prefectural Social Insurance Medical Association, Inatsuki Hospital, Kama 820-0207, Japan; 3 Department of Internal Medicine and Bioregulatory Science, Graduate School of Medical Sciences, Kyushu University, Fukuoka 812-8582, Japan; 4 Department of Health Sciences, Graduate School of Medical Sciences, Kyushu University, Fukuoka 812-8582, Japan; 5 Department of Cardiovascular Medicine, Graduate School of Medical Sciences, Kyushu University, Fukuoka 812-8582, Japan

**Keywords:** Molecular biology, Cell biology, Human metabolism

## Abstract

The occurrence of diet-induced obesity has been increasing worldwide and has become a major health concern. Mitochondria are densely distributed in brown adipose tissue and are involved in lipid consumption. Therefore, increasing energy expenditure through the activation of brown adipocytes may be a potential therapy for obesity. Our findings showed that mitochondrial transcription factor A (TFAM) homozygous transgenic (TgTg) mice had highly activated brown adipocytes and increased expression of oxidative phosphorylation, leading to resistance to obesity. Transplantation models of TFAM-expressing brown adipocytes could mimic the phenotype of TFAM TgTg mice, and proving their anti-obesity effect. We found that brown adipocytes secrete exosomes which enable self-activation in an autocrine and paracrine manner. The secretion was enhanced in TFAM TgTg brown adipocytes, resulting in a higher activation. These findings may lead to a promising treatment strategy for obesity through selective stimulation of exosome secretion.

## Highlights

• Human TFAM overexpression in BAT promotes strong anti-obesity effects
• Increasing mitochondrial function in hTFAM TgTg mice facilitates EVs secretion
• Enhanced EV released in TgTg brown adipocytes induce self-differentiation/activation

## Graphical abstract

**Figure:**
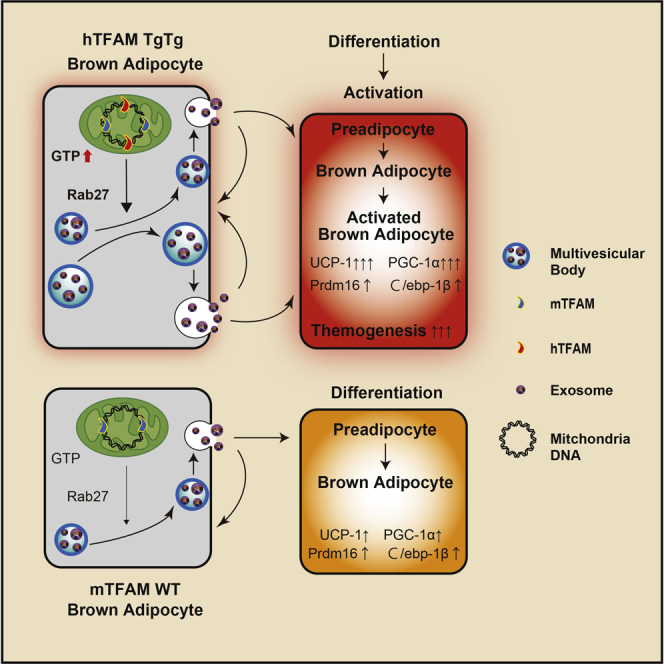

## Introduction

Obesity is expanding worldwide and is accompanied by serious medical and economic issues (Blüher, 2019). Most approved anti-obesity medications have side effects. Consequently, there is an urgent requirement for alternative therapeutic approaches (Daneschvar et al., 2016).

The brown adipose tissue (BAT) is important for energy expenditure and is mediated by tissue-specific uncoupling protein 1 (UCP-1), which is known to mediate brown adipocyte-specific nonshivering thermogenesis and energy dissipation (Lowell et al., 1993; Porter, 2006). This process modifies whole-body metabolism, insulin sensitivity, longevity, and susceptibility to weight gain (Cypess et al., 2009; Ortega-Molina et al., 2012). Because considerable amounts of BAT have been found using fluorodeoxyglucose-positron emission tomography-computed tomography (FDG-PET-CT) in adult humans (van Marken Lichtenbelt et al., 2009; Cypess et al., 2009; Saito et al., 2009; Virtanen and Al., 2009), BAT has received renewed attention. Some human and animal studies have been conducted on activated BAT over the past few decades. BAT is activated under conditions of cold- and diet-induced thermogenesis and pharmacological treatment; however, none of those studies have indicated any safe, stable, and reliable treatments for obesity (Marlatt and Ravussin, 2017).

Mitochondria are densely distributed in the brown adipocytes and provide cellular fuel through the conversion of nutrients into adenosine triphosphate (ATP) energy in the cell through oxidative phosphorylation (OXPHOS) (Gaspari et al., 2004). Mitochondrial activity and metabolite production can influence mesenchymal stem cells (MSCs), i.e., preadipocytes renewal and fate (Zhang et al., 2018; Bahat and Gross, 2019). In addition, during adipogenic cell differentiation and activation, mitochondrial DNA (mtDNA) content, mitochondrial biogenesis, respiratory enzyme complexes, oxygen consumption, and intracellular ATP content are elevated (Li et al., 2017). Thus, intervening in mitochondrial dynamics by targeting brown adipocyte differentiation and activation, thereby facilitating lipid consumption by heat production, could constitute a promising anti-obesity treatment.

Mitochondrial transcription factor A (TFAM) was firstly cloned as a transcription factor for mtDNA by Fisher and Clayton (Fisher and Clayton, 1988). TFAM was then identified as a major component of the mitochondrial nucleoid (Takamatsu et al., 2002; Alam et al., 2003) which is important for stable mtDNA maintenance (Kanki et al., 2004). Therefore, TFAM plays an essential role in mtDNA stability by regulating mtDNA replication, transcription, and packaging (Gaspari et al., 2004; Campbell et al., 2012; Kang et al., 2007).

Our previous study demonstrated that mice overexpressing human TFAM (hTFAM) under a chicken β-actin promoter were protected against cardiac failure following a myocardial infarction. Simultaneously, this genotype of mice exhibited enhanced longevity, higher glucose tolerance, and lower body weight (BW) than the wild-type (WT) mice (Ikeuchi et al., 2005). We also validated that the hTFAM transgenic mouse model highly expressed hTFAM, especially in the heart and skeletal muscle (Ikeuchi et al., 2005), although it was barely detectable in other major organs, such as the lung, liver, and kidney. However, the examination of adipose tissue has not yet been performed. Considering that the brown adipocytes develop from the myogenic regulatory factor Myf5 positive skeletal myogenic lineage (Seale et al., 2008), we examined hTFAM expression in adipose tissue and found that it was highly expressed in BAT but not in the white adipose tissue (WAT). This is consistent considering the origin of brown adipocytes.

The aim of this study was to elucidate the mechanism of TFAM-mediated weight loss. To explain the beneficial effects of TFAM modification in terms of the metabolic conditions seen in our transgenic mouse model, we focused on how BAT overexpressing hTFAM facilitates the differentiation and activity of brown adipocytes. First, we demonstrated that brown adipocytes that overexpress hTFAM played a critical role in several biological processes, including development, differentiation, and activation. Then, we found that these phenotypes were achieved via the secretion of numerous extracellular vesicles (EVs) in an autocrine and paracrine manner. This means that brown adipocytes can sustain a self-activated condition, which leads to obesity-resistance. The mechanism of EV-mediated brown adipocyte activation may provide a promising treatment strategy for obesity and its comorbidities.

## Results

### hTFAM overexpression in BAT enhances general metabolism and promotes anti-obesity effects

**Figure 1:**
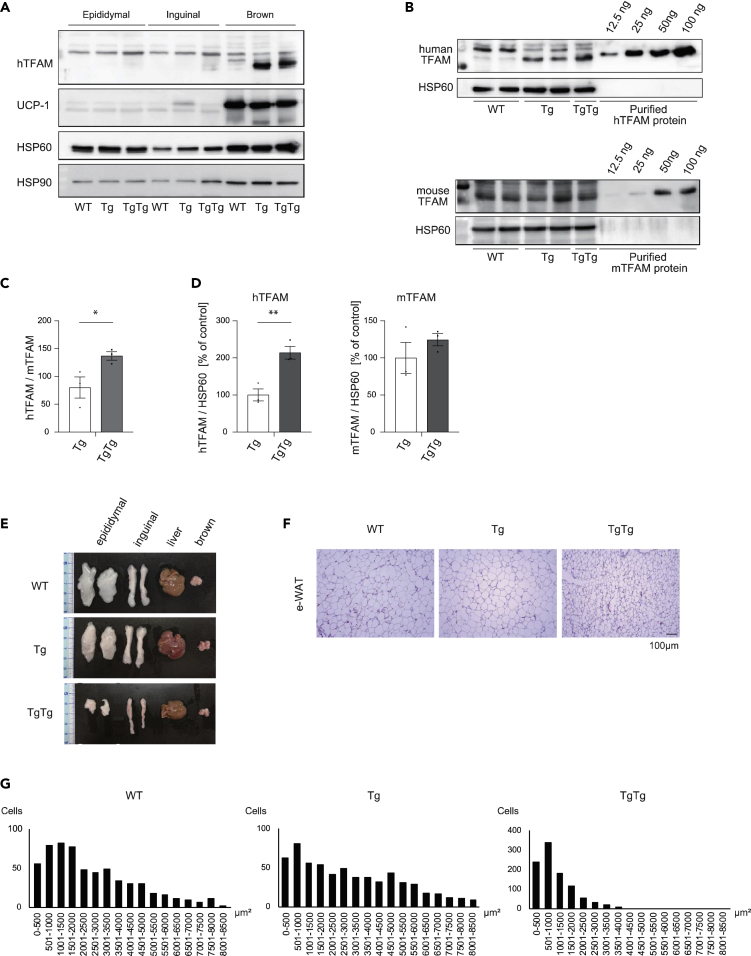
Effects of hTFAM overexpression on white and brown adipose tissue phenotypes (A) Verification of hTFAM protein expression in epididymal (e)- and inguinal (i)-WAT and BAT. (B) The h- and m- TFAM protein levels were evaluated using western blot analysis. Pictures represent western blots for h- and m- TFAM in BAT. (C) The ratio between hTFAM and mTFAM protein expression in Tg and TgTg BAT. (D) The h- and m- TFAM protein levels were normalized by the levels of HSP60. The quantitative results are expressed as the mean percentage of the level in the Tg ± standard error (SE) (n = 3/group). Total protein extracts from e− and i-WAT and BAT probed with a polyclonal antiserum against h- and m- TFAM. (E) Photographs show representative conditions of e − and i-WAT, liver, and BAT from NCD-fed WT, Tg, and TgTg mice. (F) Effect of hTFAM overexpression on e-adipocyte size. Pictures are representative of each sample stained with hematoxylin and eosin and examined by optical microscopy. Scale bar represents 100 μm. (G) Frequency distribution of the e-adipocyte area in NCD-fed WT, Tg, and TgTg mice. ∗∗p < 0.01, versus Tg mice. Two-tailed unpaired Student’s *t* test in (C) and (D).

We initially measured hTFAM expression in BAT and WAT. The expression of hTFAM which is controlled by the chicken β-actin promoter, was expressed mainly in brown adipocytes (Figure 1A), which is consistent with their origin from the Myf5 positive skeletal myogenic lineage (Seale et al., 2008). The detected proteins were confirmed by purified hTFAM and mouse (mTFAM) proteins. hTFAM protein expression was approximately 1.33 fold higher than endogenous mTFAM expression in homozygous (TgTg) mice (Figures 1B and 1C). Therefore, the total amount of TFAM (hTFAM 1.33; mTFAM 1.0) was 2.33 higher compared to the physiological condition. Furthermore, hTFAM expression was approximately 2.1 times higher in TgTg mice than in heterozygous (Tg) mice (Figure 1D).

Next, we determined the characteristics of hTFAM TgTg mice under a normal chow diet (NCD) for 24 weeks. BW was significantly lower in the TgTg mice compared to the WT as was the weight of inguinal (i)- and epididymal (e)-WAT. On the other hand, the weight of the BAT normalized with the size of the tibia was significantly decreased to 63.5% compared to the WT, however, the degree of decline was more gradual compared to that in the i- and e-WAT (Figures 1E and S1) (Table 1). Histological analysis showed that TgTg promoted a reduction in the adipocyte area, which is consistent with the decreasing weight of i- and e-WAT in TgTg mice (Figures 1F and 1G). In the case of Tg, no anti-obesity effect was observed.

**Table 1:** Characteristics of WT, Tg, and TgTg mice after 24 weeks on a normal chow diet (NCD)

	WT	Tg	TgTg	TgTg (%)
BW (g)	40.4 ± 0.46	38.1 ± 0.43	25.3 ± 0.64^###^	
e − WAT (mg)	2123.3 ± 101.1	1446.7 ± 27.3	356.7 ± 8.82^###^	20.37 ± 20.64^###^
i – WAT (mg)	790.0 ± 26.5	660.0 ± 45.1	226.7 ± 17.5^###^	35.0 ± 2.77^###^
BAT (mg)	156.7 ± 8.82	153.3 ± 3.3	83.3 ± 3.3^###^	65.31 ± 2.22^a^
Liver (mg)	1816.7 ± 72.7	1846.7 ± 27.3	1423.3 ± 43.4^a^	96.19 ± 1.56

TgTg(%): tissue weight/tibia (% of control).

a p < 0.01, ^###^p < 0.001, versus WT. One-way ANOVA with Turkey’s multiple comparisons test.

**Figure 2:**
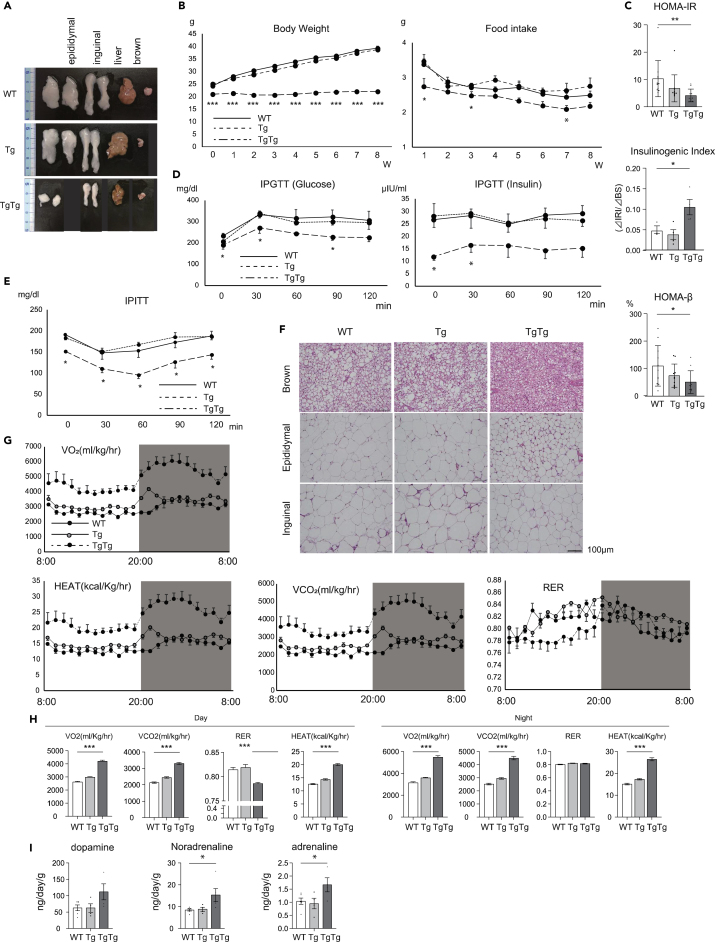
Metabolic characterization in WT, Tg, and TgTg mice under a HFD (A) TgTg mice had extremely low e− and i-WAT accumulation on the HFD compared to WT mice. (B) Changes in body weight and food intake (n = 5–10/group). (C) Evaluation of insulin resistance (homeostasis model assessment of insulin resistance: HOMA-IR) and β cell function (insulinogenic index, homeostasis model assessment of beta-cell function: HOMA-β) (n = 5–10/group). (D) Glucose tolerance in 16 h-fasted mice after an intraperitoneal injection of glucose (5 mg/kg body weight); blood glucose and insulin levels measured at the indicated time points (n = 4–6/group). (E) Insulin sensitivity in 16 h-fasted mice after an intraperitoneal insulin tolerance test (0.5 units/kg body weight) (n = 4–6/group). (F) Microscopic e− and i-WAT and BAT sections stained with hematoxylin and eosin. Scale bars represent 100 μm. (G and H) VO_2_, VCO_2_, heat production, and RER were measured using an open-cage calorimetry system (n = 3/group). (I) Urinary levels of catecholamine, dopamine, noradrenalin, and adrenalin determined by high-performance liquid chromatography-mass spectrometry (HPLC-MS) (n = 4–6/group). ∗p < 0.05, ∗∗p < 0.01, ∗∗∗p <0.001, versus WT mice. One-way ANOVA with Turkey’s multiple comparisons test in (B) – (E), (H) and (I). Bar graphs represent mean ± SE.

Eight-week-old mice were then fed a high fat diet (HFD) for 8 weeks. TgTg mice had extremely low i- and e-WAT accumulation compared with the WT mice (Figure 2A). The weight of i- and e-WAT was lower in TgTg mice, however, the ratio of BAT to the size of the tibia showed no difference between WT and TgTg mice (Table 2). Moreover, TgTg mice showed no weight change over the 8 weeks, even though the amount of intake was almost the same as that in the WT group (Figures 2B and S2F). In addition, insulin resistance and glucose tolerance improved significantly in TgTg mice (Figures 2C–2E). Histological analysis showed lipid accumulation in BAT in mice on the HFD in the WT group; however, the lipid accumulation did not occur in the BAT of the TgTg mice (Figure 2F).

**Table 2:** Characteristics of WT, Tg, and TgTg mice after 8 weeks on a HFD

	WT	Tg	TgTg	TgTg (%)
BW (g)	36.4 ± 1.6	35.9 ± 1.41	22.0 ± 0.56^###^	
e − WAT (mg)	2166.3 ± 132.3	2048.8 ± 79.7	465.7 ± 65.9^###^	44.57 ± 4.63^###^
i – WAT (mg)	1333.8 ± 88.6	1178.8 ± 117.3	332.9 ± 35.0^###^	52.34 ± 4.07^###^
BAT (mg)	160 ± 18.0	148.8 ± 17.1	68.6 ± 6.7^###^	91.55 ± 7.30
Liver (mg)	1580 ± 180.8	1427.5 ± 146.0	1030.0 ± 35.9^a^	141.17 ± 3.13^###^

TgTg(%): tissue weight/tibia (% of control).

a p < 0.05, ^###^p < 0.001, vs WT. One-way ANOVA with Turkey’s multiple comparisons test.

**Figure 3:**
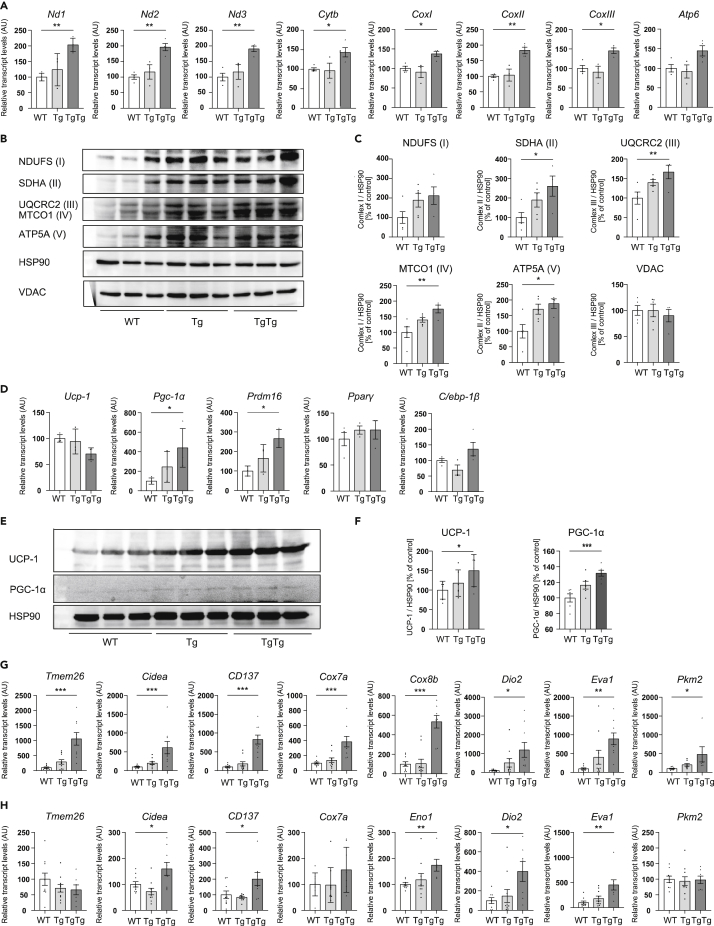
Effects of hTFAM overexpression on BAT activation and WAT browning (A) Mitochondrial OXPHOS mRNA expression in brown adipose tissue. Total RNA was extracted from HFD-fed WT, Tg, and TgTg mice. All mRNA levels were measured by real-time RT-PCR. The mRNA levels were normalized to the levels of hypoxanthine-guanine phosphoribosyltransferase (*Hprt*), and the results are expressed as the mean percentage of the level in control WT mice ±SE (n = 3–4/group). (B) OXPHOS protein levels were evaluated using western blot analysis. Pictures represent western blots for OXPHOS in BAT and OXPHOS levels were normalized by the level of HSP90. (C)The quantitative results are expressed as the mean percentage of the level in the WT ± SE (n = 4–5/group). (D) Thermogenic and brown adipocyte differentiation/activation-related gene expression in BAT, as assessed by RT-PCR. (E and F) UCP-1 and PGC-1α protein levels in BAT were evaluated by western blot analysis (n = 4–9/group). The mRNA levels of beige adipocyte markers in i-WAT (G) and e-WAT (H) were assessed by RT-PCR (n = 7–10/group). ∗p < 0.05, ∗∗p < 0.01, ∗∗∗p < 0.001, versus WT mice. One-way ANOVA with Turkey’s multiple comparisons test in (A), (C), (D) and (F) – (H). Bar graphs represent mean ± SE.

Analysis of the metabolic rate showed that oxygen consumption rates (VO_2_) and carbon dioxide production rates (VCO_2_) were increased in TgTg mice, both during the day and night (Figures 2G and 2H). Furthermore, the respiratory exchange ratio (RER) was significantly lower in TgTg mice (Figures 2G and 2H), which is consistent with the acceleration of lipid metabolism because of increasing brown adipocyte markers in both i- and e-WAT (Figures 3G and 3H and S3C–S3H). On the other hand, heat production was increased through BW normalization, however, there was no significant change without BW normalization. Nevertheless, the deep body temperature (rectal temperature) tended to increase in TgTg (Figure S3J), indicating that hTFAM overexpression may contribute to facilitate heat production.

The daily production of dopamine, noradrenalin, and adrenalin were examined by urine collection and adjusted by the total urine volume and BW. Noradrenaline and adrenaline production was significantly increased in TgTg mice (Figure 2I). These circulating catecholamines could mediate the inter-organ network between BAT and i- and e-WAT.

The ratio of each limb muscle weight to total BW (Figure S2A) showed that the soleus muscle, a Type I fiber rich in mitochondria and capillaries conducting OXPHOS to generate energy (Schiaffino and Reggiani, 2011), was bigger in TgTg mice, and the gastrocnemius (thigh) muscle, a Type II fiber with low mitochondrial content (Schiaffino and Reggiani, 2011), was smaller in the TgTg mice compared to the WT. In contrast, the concentrations of mitochondrial OXPHOS proteins in Type II fibers were significantly higher in complex I, III, and V fibers (Figures S2D and S2E), whereas almost no change was observed in Type I fibers (Figures S2B and S2C).

Collectively, these results indicated that hTFAM overexpression in BAT significantly increased metabolic activity while maintaining initial weight and avoiding lipid accumulation in systemic organs under HFD feeding conditions.

### hTFAM promotes BAT mitochondrial OXPHOS, resulting in both activation of brown adipocytes and browning of white adipocytes

To examine the effect of hTFAM overexpression on mitochondrial enzymes involved in mitochondrial OXPHOS in BAT, mRNA and protein expression of each complex was analyzed. qPCR analysis showed that the expression in TgTg was significantly higher than in the WT (Figure 3A). Although the protein expression of the voltage-dependent anion channel (VDAC) showed no difference between WT and TgTg, complexes II–V were significantly increased in TgTg (Figures 3B and 3C), indicating that mitochondrial activity in brown adipocytes was accelerated by hTFAM overexpression. In parallel to increasing mitochondrial activity, UCP-1 protein expression increased significantly, as did deep body temperature in TgTg mice (Figures 3E and 3F and S3J). Furthermore, PPARγ-coactivator-1α (*Pgc-1α*), and PR domain containing 16 (*Prdm16*) mRNA were significantly increased (Figure 3D).

Of note, mitochondrial OXPHOS mRNA expression increased significantly in the remote i- and e-WAT (S Figures 3A and 3B), although hTFAM was not overexpressed in these tissues (Figure 1A). Furthermore, the transcription factor that white adipocytes need for browning was significantly upregulated (Figures S3C and S3F). With the increased expression of the transcription factors, the mRNA levels of beige-specific genes were also elevated (Figures 3G and 3H). Consistent with the upregulated browning and large decrease in the tissue weight of i- and e-WAT, the mRNA (Figures S3C and S3F) and protein (Figures S3D and S3E and S3G and S3H) levels of UCP-1 were significantly increased.

In this way, appropriately promoted mitochondrial function in brown adipocytes through hTFAM overexpression, induced both thermogenesis and brown adipocyte differentiation/activation-related mRNA and proteins, and WAT browning maker mRNA and proteins, which was reflected in the higher deep body temperature (Figure S3J) as well as in the weight loss (Figure 2B).

### Effects of hTFAM overexpressed primary brown adipocyte transplantation in HFD fed WT mice

**Figure 4:**
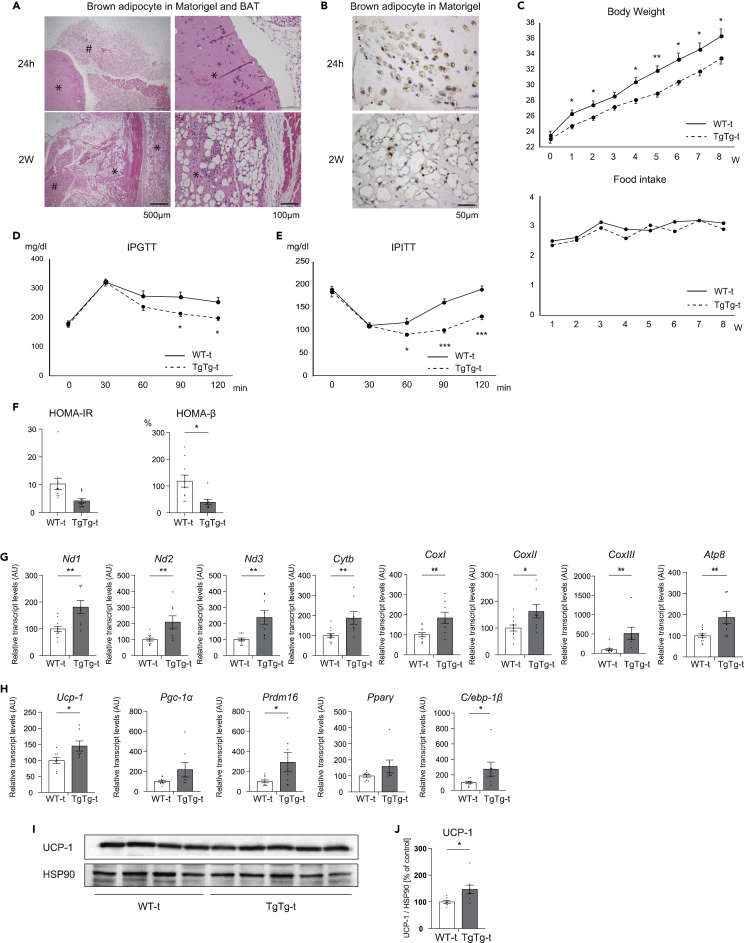
Effects of hTFAM-overexpressing brown adipocyte transplantation on metabolic characterization, BAT activity, and WAT browning in HFD-fed mice (A) Activated hTFAM-overexpressing brown adipocytes were injected subcutaneously around the BAT. Matrigel was confirmed around the BAT as a white substance. Pictures are representative of the Matrigel on the perimeter of the BAT at each time point (24 h and 14 d after injection); stained with hematoxylin and eosin. (# BAT, ∗Matrigel). Scale bars represent 500μm and 100 μm. (B) Immunostaining analysis of hTFAM-overexpressing brown adipocytes in Matrigel at each time point. Scale bar represents 50 μm. (C) Changes in body weight and food intake (n = 6–11/group). (D) Glucose tolerance was determined in 16 h-fasted mice after an intraperitoneal injection of glucose (5 mg/kg body weight) (n = 8–10/group). (E) Insulin sensitivity was determined in 16 h-fasted mice after an intraperitoneal insulin tolerance test (0.5 units/kg body weight) (n = 8–10/group). (F) Evaluation of insulin resistance (HOMA-IR) and β-cell function (HOMA-β) (n = 8–10/group). (G) Mitochondrial OXPHOS and (H) thermogenic and brown adipocyte differentiation/activation-related gene expression in BAT. All mRNA levels were measured by RT-PCR. The mRNA levels were normalized to the levels of *Hprt*, and the results are expressed as the mean percentage of the level in WT-t control mice ±SE (n = 7–11/group). UCP-1 protein levels in BAT were evaluated by western blot analysis (I and J) (n = 8–9/group).∗p < 0.05, ∗∗p < 0.01, ∗∗∗p < 0.001, versus WT - t. Two-tailed unpaired Student’s *t* test in (C) – (H) and (J). Bar graphs represent mean ± SE.

To understand the anti-obesity effect of hTFAM-overexpressing TgTg brown adipocytes alone, adipocytes originating in hTFAM-overexpressing mice were transplanted into WT mice. First, we determined whether the harvested brown preadipocytes differentiated into mature brown adipocytes after incubation through induction and maintenance media (Figures S4A and S4B). Brown adipocytes (Figures S4C and S4D) kept in the Matrigel were injected beside the BAT in recipient WT mice. Because the transplanted cells grafted well, which was confirmed by hematoxylin and eosin (HE) staining and immunohistochemistry analysis (Figures 4A and 4B), we fed recipient mice a HFD for 8 weeks. As expected, BW was significantly lower in TgTg transplanted mice (TgTg-t) compared to WT transplanted mice (WT-t) under the same food intake condition (Figure 4C) (Table 3), with significant improvement in insulin resistance and glucose tolerance (Figures 4D–4F). The weight of i- and e-WAT was also significantly decreased in TgTg-t mice, however, the real BAT weight and the ratio of BAT to the size of the tibia showed no difference between TgTg-t and WT-t mice (Table 3).

**Table 3:** Characteristics of WT-t and TgTg-t mice after 8 weeks on an HFD

	WT - t	TgTg - t	TgTg – t (%)
BW (g)	36.3 ± 1.47	31.5 ± 0.7^a^	
e − WAT (mg)	1764 ± 101.5	1311.3 ± 123.7^##^	76.09 ± 7.19^a^
i – WAT (mg)	1178 ± 98.0	730.0 ± 75.7^##^	63.36 ± 6.59^##^
BAT (mg)	118 ± 13.0	100.0 ± 8.5	86.37 ± 7.64
Liver (mg)	1566 ± 119.6	1206.3 ± 28.7^a^	79.06 ± 2.22^a^

TgTg(%): tissue weight/tibia (% of control).

a p < 0.05, ^##^p < 0.01, vs WT. Two-tailed unpaired Student’s *t* test.

Next, we investigated mitochondrial OXPHOS in the BAT. The OXOPHOS mRNA expressions were significantly increased (Figure 4G) in parallel with *Ucp-1*, *Prdm16*, and *C/ebp-1β* mRNA and UCP-1 protein expression (Figures 4H and 4I and 4J) in endogenous TgTg-t BAT. Although the i- and e-WAT are distant from the brown adipocyte-transplanted site, the weight of the tissues was also significantly decreased. In addition, both i- and e-WAT mitochondrial OXPHOS mRNA expressions were significantly increased in TgTg-t mice (Figures S4E and S4F). Furthermore, the transcription factors needed for the browning of white adipocytes were increased (Figures S4G and S4I) and, as a result, most of the browning markers in both i- and e-WAT, including UCP-1 protein expression, were significantly increased (Figures S4H and S4J) (Figures S5A and S5B), which is consistent with the reduction in WAT weight and upregulation of mitochondrial activity. Moreover, histological analysis showed that TgTg promoted a reduction in the adipocyte area in both the BAT and WAT (Figure S5C).

These results suggested that the transplanted active brown adipocytes possess anti-obesity effects by activating brown adipocytes in the recipient. They also remotely induced a qualitative shift from white to beige adipocytes. These observations indicated that obesity resistance in TgTg-t is directly attributable to activated hTFAM overexpressing brown adipocytes.

### TgTg brown adipocyte differentiation and activation by humoral factors in an autocrine or paracrine manner

**Figure 5:**
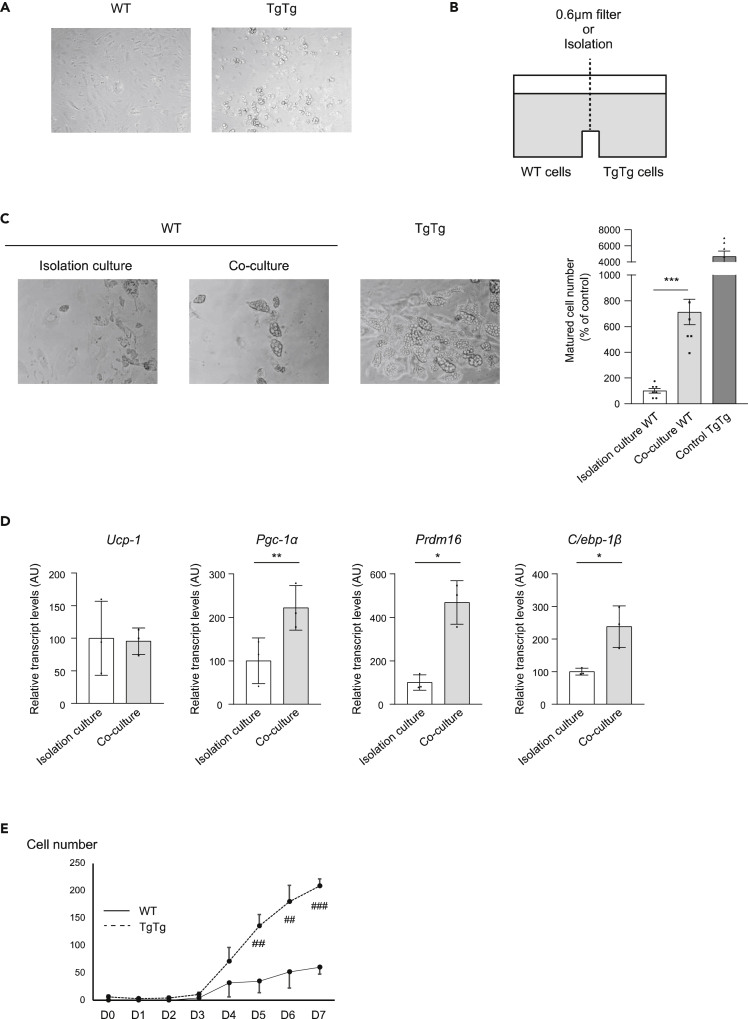
TgTg adipocytes secrete humoral factors and increase brown adipocyte activity (A) Spontaneous adipocyte differentiation in TgTg cells incubated with regular medium for 7 d. (B) Cross-section of the co-culture well. (C) Differentiated positive control TgTg adipocytes and aspects of WT adipocytes incubated in isolation or co-culture well. Number of mature cells was counted, and the results were expressed as the mean percentage of the number in WT adipocytes incubated in the isolation culture ± SE. ∗∗∗p< 0.001, versus isolation culture. (D) Brown adipocyte differentiation/activation-related gene expression assessed by RT-PCR. Levels of mRNA were normalized to the levels of *Hprt*, and the results are expressed as the mean percentage of the level in WT adipocytes incubated in the isolation culture ±SE (n = 3/group). ∗p < 0.05, ∗∗p< 0.01, versus isolation culture. (E) Differentiated WT cell numbers in the supernatants, transplanted from TgTg cell culture medium (n = 6/group). ^##^p < 0.01, ^###^p < 0.001, versus WT supernatants. One-way ANOVA with Turkey’s multiple comparisons test in (C). Two-tailed unpaired Student’s *t* test in (D) and (E). Bar graphs represent mean ± SE.

To reveal the mechanism of obesity resistance and the remote effects on low-hTFAM-expressing adipose tissues in TgTg-t, we observed the change in characteristics of the primary brown preadipocyte culture over time without stimuli for differentiation. Notably, when we did not change the medium for a week, the primary preadipocytes originating in TgTg, differentiated into mature adipocytes, identifiable by multiple lipid droplets, which did not occur in the WT (Figure 5A). Next, we co-cultured the WT and TgTg preadipocytes by separating them with a 0.6 μm filter or non-permeable sheet (Figure 5B). The WT cells partly differentiated and grew multiple lipid droplets similar to the TgTg cells after 7 days of seeding the cells, however, the completely separated WT cells showed almost no differentiation (Figure 5C). We also examined brown adipocyte differentiation- and activity-related gene expression. The 0.6 μm filtered WT cells showed significantly higher expression of *Pgc-1α*, *Prdm16*, and *C/ebp-1β*, compared to isolated WT cells (Figure 5D). We then observed the WT cell phenotype after adding cell culture supernatants of WT or TgTg. Although we observed that a small number of WT cells differentiated with WT supernatants, more cells showed significant differentiation in the TgTg supernatant group (Figure 5E). These data indicated that TgTg cells secrete substances that induce adipocyte differentiation and activation in an autocrine or paracrine manner.

### Enhanced EV released in the TgTg brown adipocytes accelerate differentiation and activity

**Figure 6:**
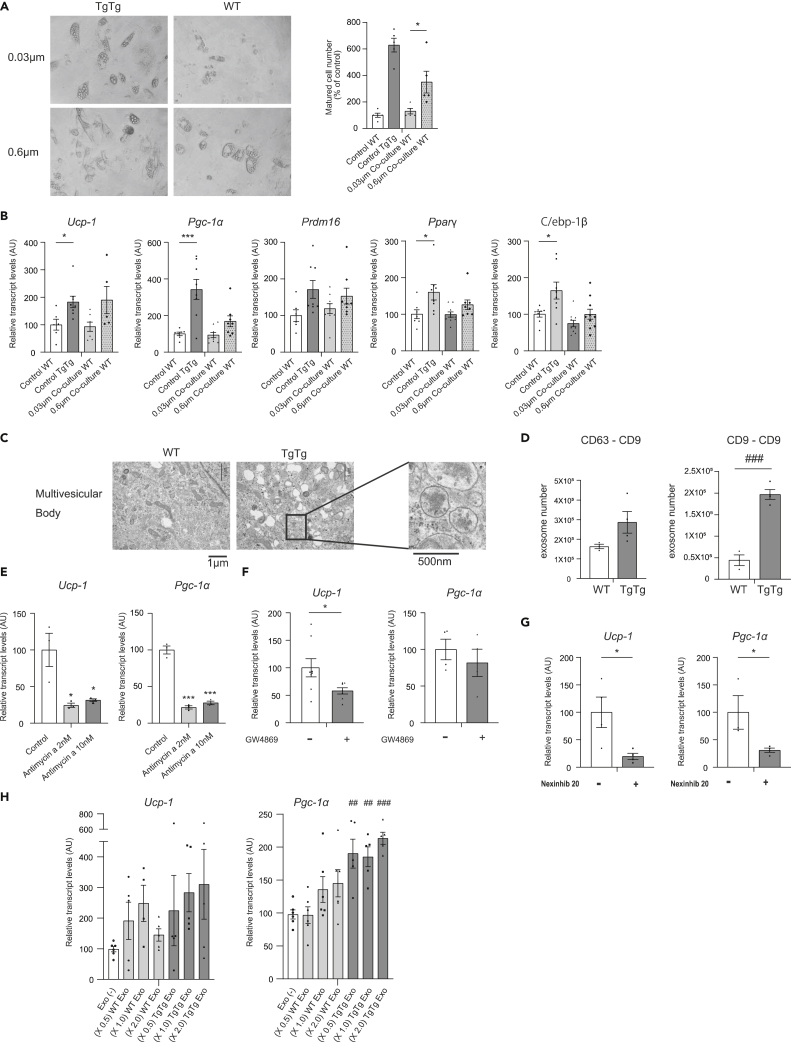
Enhanced EV release in TgTg brown adipocytes increases differentiation and activity (A) Differentiated positive control TgTg preadipocytes and aspects of WT preadipocytes incubated in 0.03 μm or 0.6 μm filtered co-culture well. Number of mature cells was counted, and the results were expressed as the mean percentage of the number in control WT adipocytes incubated in the isolation culture ±SE. ∗p < 0.05, versus 0.6 μm co-culture WT. (B) Thermogenic and brown adipocyte differentiation/activation-related gene expression in BAT, as assessed by RT-PCR. The mRNA levels were normalized to the levels of *Hprt*, and the results are expressed as the mean percentage of the level in control WT adipocytes incubated in the isolation culture ±SE (n = 10/group). ∗p < 0.05, ∗∗∗p < 0.001, versus control WT. (C) Molecular features of WT and TgTg brown adipocytes, assessed by electron microscopy. Scale bars represent 1 μm and 500 nm. High-power view of TgTg shows the MVB (boxed area). (D) Quantification of EVs derived from WT or TgTg brown adipocytes with the ExoCounter using anti-CD63 (left) or anti-CD9 (right) Ab-coated discs and beads conjugated with Ab against CD9 (n = 4/group). ^###^p < 0.001 versus WT. Inhibitory effect of exosomes on TgTg brown adipocytes incubated with or without (E) Antimycin A, (F) GW4869, or (G) nexinhib20. The expression of genes related to brown adipocyte activation was assessed by RT-PCR (n = 3/group) (E), (n = 4–8/group) (F, G). ∗p < 0.05, ∗∗∗p < 0.001 versus Antimycin A (−), GW4869(−), or Nexinhib(−). (H) Brown adipocyte activation-related gene expression in adipocytes cultured with EVs purified from WT and TgTg adipocytes, assessed by RT-PCR (n = 5/group). ^##^p < 0.01, ^###^p < 0.001 versus Exo(−). Two-tailed unpaired Student’s *t* test in (D), (F) and (G). One-way ANOVA with Tukey’s multiple comparisons test in (A), (B), (E), and (H). Bar graphs represent mean ± SE.

To explore the cause of enhanced auto-activation in the TgTg brown adipocytes, we performed a co-culture study in which WT and TgTg cells were separated by 0.6 μm or 0.03 μm pore size filters. Our results found that WT adipocytes exhibited almost no cell differentiation when the well was separated by 0.03 μm (Figure 6A). Meanwhile, cells separated by the 0.6 μm filter did exhibit differentiation. Consistent with the cell phenotype, 0.03 μm filtered WT cells did not show brown adipocyte differentiation and activation, but 0.6μm filtered WT cells had a tendency to control TgTg preadipocytes, as shown by mRNA expression of *Ucp-1*, *Pgc-1α*, *Prdm16*, *Pparγ*, and *C/ebp-1β* (Figure 6B).

The finding that adipocyte differentiation and activation were blocked by the 0.03 μm filter placed the focus on EVs, especially exosomes, with a diameter of 0.03–0.15 μm (30–150 nm). Transmission electron microscopy (TEM) revealed that multivesicular bodies (MVBs) in TgTg cells were much more abundant than in WT cells (Figure 6C). We then counted the EVs derived from the WT and TgTg cells and analyzed them using an ExoCounter. Using anti-CD9 Ab (disc) and anti-CD63 (beads), CD9/CD63 double-positive EVs were detected in cells of both genotypes, with higher concentrations seen in TgTg cells. Moreover, using anti-CD9 Ab (disc and beads), significantly more EVs occurred in TgTg cells (Figure 6D). The diameter of the detected EVs, analyzed by NanoSight, was between 50–150 nm, consistent with exosome size (Figures S7A and S7B).

We examined nucleotide production in cells of both genotypes. All nucleotides were significantly overexpressed in TgTg cells, suggesting a higher energy metabolism state in TgTg than in WT cells (Figure S8). Antimycin A, an inhibitor of complex III, strongly suppressed the expression of *Ucp-1* and *Pgc-1α* (Figure 6E), which suggests that the enhanced OXOPHOS activity caused by hTFAM-expression is essential for the activation.

GW4869, which inhibits the ceramide-mediated inward budding of MVBs and therefore the release of mature exosomes (Essandoh et al., 2015), and nexinhib20, an inhibitor of the interaction between Rab27a and its effector, are known to cause the downregulation of MVB docking to the plasma membrane and exosome secretion (Johnson et al., 2016; Song et al., 2019). Both GW4869 (Figure 6F) and nexinhib20 (Figure 6G) showed decreased expressions of brown adipocyte activation marker genes. The inhibitory effect of nexinhib20 was much stronger than GW4869. Moreover, we confirmed the direct effect of EVs released from WT and TgTg adipocytes on adipocyte activity. We found that the EVs from both genotypes increased the expression of activation marker genes (Figure 6H). *Pgc-1α* mRNA expression especially, was increased in a dose dependent manner. Furthermore, *Ucp-1* mRNA expression showed partial dose dependency. In addition, we evaluated the contents of the EVs from both WT and TgTg adipocytes using secretome analysis. There was no major difference in the exosome content, which contribute to brown adipocyte differentiation and activation (Table S1), suggesting that EVs per se are essentially the same between WT and TgTg. Therefore, the secretion capacity of EVs rather than their content may be the decisive factor in brown adipocyte activity regulation.

Finally, to confirm whether the EVs are responsible for the WAT browning, we used the co-culture (TgTg brown adipocytes and differentiated 3T3-L1 cells) with same time course as brown adipocyte study. We found that the browning marker gene expression, *Ucp-1*, *Cd137*, and *Eva1*, are increased in co-culture 3T3-L1 cells (Figures S5D and S5E). This means that EVs may influence, at least in part, white adipocyte thermogenesis and its characteristics.

Collectively, these results suggest that a higher mitochondrial function in hTFAM-overexpressing brown adipocytes facilitates MVB traffic and EV release, resulting in the activation of brown adipocytes. Moreover, the beneficial effects of EVs occurred in a dose-dependent manner.

## Discussion

Obesity is associated with reduced BAT function, as evidenced by lipid accumulation and mitochondrial dysfunction and loss (Hung et al., 2014; Shimizu et al., 2014). It has also been reported that the downregulation of the OXPHOS system in WAT is correlated with the level of obesity (De Pauw et al., 2009; Kaaman et al., 2007; Heinonen et al., 2015). In the present study, we showed that hTFAM overexpression in the BAT of mice exhibited marked resistance against age- and diet-induced obesity, produced higher energy expenditure, improved insulin secretory function and resistance, and increased mitochondrial function. We also found that hTFAM-overexpressing brown adipocytes release more EVs which work through an autocrine or paracrine manner, resulting in a virtuous cycle, thereby maintaining BAT activity and exhibiting anti-obesity effects.

Several TFAM expression-modified animal model studies involving metabolism have been reported. There are reports showing that TFAM deletion in adipose tissue (Vernochet et al., 2012) and overexpression in skeletal muscle (Koh et al., 2019) increased mitochondrial function through remodeling the OXPHOS complexes heterogeneously, exhibited anti-obesity effects, and improved general metabolic improvement. Contrary to the findings described in the report above (Vernochet et al., 2012). TFAM downregulation in adipose tissue led to adipocyte death and whitening of BAT (Vernochet et al., 2014), with decreased expression and enzymatic activity of the OXPHOS system because TFAM is essential for mtDNA transcription and replication. In the present study, hTFAM overexpression in BAT (TgTg) resulted in increased mitochondrial function through the comprehensive remodeling of the OXPHOS complexes (Figures 3A–3C), however, there was no effect in the hTFAM heterozygous (Tg) mice in terms of mitochondrial function and obesity resistance (Figures 2A and 2B and 3A–3C), presenting a phenotype similar to that of the WT. It has been reported that a mild increase in TFAM levels (∼2-fold) leads to dramatic functional effects on transcription and mtDNA replication (Farge et al., 2014), whereas forced overexpression of TFAM results in mtDNA depletion and reduced enzymatic activity (Farge et al., 2014; Kukat et al., 2015; Ylikallio et al., 2010). As seen in our results, the BAT in TgTg mice had approximately 1.33 times higher TFAM expression than that of endogenous mTFAM (the total expression of TFAM was 2.33 fold higher compared to the physiological condition, adding hTFAM [1.33] and mTFAM [1.0]). A 2.33-fold TFAM expression may be appropriate to elicit higher mitochondrial activity leading to BAT activity, phenotypic obesity resistance, and general metabolic improvement.

In our hTFAM transgenic mice, hTFAM is highly expressed in the heart and skeletal muscle (Ikeuchi et al., 2005), but barely detected in other major organs, such as the lungs, liver, and kidneys. Because brown adipocytes develop from a Myf5 positive skeletal myogenic lineage (Seale et al., 2008), the mice also exhibited strong hTFAM expression in BAT but very low levels in WAT (Figure 1A). Furthermore, in this mouse model, OXPHOS expression in slow-twitch muscles, such as the soleus muscle, which is rich in mitochondria and capillaries (Schiaffino and Reggiani, 2011), did not significantly differ between WT and TgTg mice (Figures S2B and S2C), whereas in fast-twitch muscles, such as the extensor digitorum longus, with low mitochondrial content (Schiaffino and Reggiani, 2011), complex I, III, and V showed increased expression (Figures S2D and S2E).

To eliminate all metabolic effects in skeletal muscle, we performed an *ex vivo* cellular experiment (Min et al., 2016; White et al., 2019). We extracted brown adipocytes from mice and re-implanted them into donors. We then evaluated the independent anti-obesity ability of TFAM-overexpression in brown adipocytes. We first determined the appropriate brown adipocyte number for transplantation; 1×10^6^ cells, a relatively smaller number compared to previous reports (Min et al., 2016; Kishida et al., 2015), were decided as suitable for graft survival and optimal weight loss. The solidified Matrigel in the circumference of BAT did not elicit immunoreaction, i.e., there was no accumulation of immune cells, in any time course, between 24 h and 2 weeks after transplantation (Figure 4A). We also confirmed that the transplanted adipocytes had matured during the second week, as exemplified by multiple lipid droplets and an integrating well in the surrounding tissue (Figure 4B).

TgTg mice showed significant weight loss on the HFD, mainly via i- and e-WAT loss (Table 2), despite TFAM not being overexpressed in WAT (Figure 1A). However, the gene expression of OXPHOS and beige adipocyte markers were increased in WAT, which is similar to the phenomenon induced by sustained thermogenic activation (Villarroya et al., 2017). Compared to BAT, mitochondria in WAT are fewer in number, smaller in size, contain fewer cristae, and have lower expression levels of fatty acid β-oxidation-related enzymes and acyl CoA dehydrogenase, resulting in triglyceride storage (Cedikova et al., 2016). However, the “brown-like” cells (also called “beige” cells) within white adipose depots are not derived from the Myf-5 lineage (Seale et al., 2011; Wu et al., 2012), but possess high mitochondrial content and OXPHOS capacity (Ohyama et al., 2016). The conversion of white into beige adipocytes is typically induced by certain environmental cues, such as β2 or 3-adrenergic receptor stimulus by catecholamine, chronic cold exposure, exercise, or long-term treatment with a PPARγ agonist (Vosselman et al., 2012; Yamaga et al., 2008; Kuji et al., 2008; Harms and Seale, 2013). In the present study, the absolute daily catecholamine production did not differ between WT and TgTg mice, even though the BW was significantly lower in TgTg mice, indicating that the catecholamine levels per unit volume of adipose tissue were significantly higher in TgTg (Figure 2I).

Recently, an inter-tissue communication mechanism mediated by EVs containing nicotinamide phosphoribosyl transferase (NAMPT) has been suggested. NAMPT is a key biosynthetic enzyme of NAD^+^ that induces activation of sirtuins. NAMPT-containing EVs secreted from both white and brown adipocytes internalize into primary hypothalamic neurons, resulting in the activation of the sympathetic nervous system (Imai, 2016; Yoshida et al., 2019; Tokizane and Imai, 2021). In this article, we showed that EVs from brown adipocytes enhanced the differentiation and activation of WT cells (Figure 6H). In addition, we showed that hTFAM-overexpressing brown adipocytes secrete more EVs than the WT cells (Figure 6D). These reports and our results clearly link an important key concept explaining the mechanism sustaining high catecholamine production levels and activation of brown adipocytes. It is however still unclear whether it is the same EVs and intra-vesicular factors responsible for the two phenomena mentioned above.

The fact that hTFAM-overexpressing brown adipocytes accelerate EVs secretion prompts us to question the relationship between brown adipocyte activation and remote effects on i- and e− WAT browning (Figures 3G and 3H) (Figures S3C–S3H) (Figures S4G–S4J). During the co-culture study, TgTg brown adipocyte and differentiated 3T3-L1 cells showed that only some browning marker gene expression was increased (Figures S5D and S5E). Nevertheless, these data indicated that EVs partially contribute to thermogenesis even in white adipocytes and affect the white adipocyte characteristics. Therefore, it is expected that i- and e− WAT browning may go through the process with other factors such as catecholamine *in vivo*.

The overexpression of all nucleotides in TgTg cells, (Figure S8) and the inhibitory effects of antimycin A on the expression of *Ucp-1* and *Pgc-1α* (Figure 6E) suggests that the higher exosome secretion and enhanced differentiation depend on the higher OXOPHOS activity caused by hTFAM-expression. Moreover, both GW4869 and nexinhib20 suppress the expression of brown adipocyte activation marker genes (Figures 6F and 6G). Of note, the inhibitory effect of nexinhib20 was much stronger than that of GW4869. Because Rab-GTPase is associated with vesicles and the inner side of the plasma membrane and participates in intracellular trafficking of vesicles (Jordens et al., 2005; Blanc and Vidal, 2018), increased GTP levels in hTFAM brown adipocytes might enhance the switch from Rab to the active state on binding of GTP (Blanc and Vidal, 2018), resulting in EV secretion.

In addition, it is reported that EVs released by BAT contain miR-99b accumulated in hepatocytes and reduced hepatic FGF21 levels, resulting in decreased circulating FDF21 (Thomou et al., 2017). In this study, serum FGF21 levels in both TgTg and TgTg-t were significantly lower than those in the control (Figure S3I) (Figure S6). Together with more EVs released from hTFAM-overexpressing brown adipocytes, this is consistent with the mechanism by which exosomal miRNAs derived from BAT regulate whole-body metabolism (Thomou et al., 2017). In the *in vitro* study, we determined that primary brown adipocytes from TgTg mice have potent differentiation and activation abilities without a preadipogenic medium (Figure 5A).

The 0.6 μm-filtered co-culture showed that the differentiation-related genes in WT adipocytes were significantly increased compared to those in the completely separated culture (Figure 5D). In addition, WT adipocytes cultured in the conditioned medium originating in the TgTg adipocyte culture increased differentiation compared to the regular medium (Figure 5E). These data led us to the hypothesis that an autocrine or paracrine mechanism accelerates brown adipocyte differentiation and activity. Because the size of the candidate substance is over 30 nm (Figures 6A and 6B), we speculate that exosomes, which are a class of EVs that range between 30 to 150 nm in size and are formed from MVBs along the endocytic pathway (Quan and Kuang, 2020), play an important role in regulating brown adipocytes. This speculation is supported by the exosome inhibition study with GW4869 (Figure 6F) and nexinhib20 (Figure 6G).

The activation of adipocytes undergoes a subsequent multi-step activation of the transcription factor network, which affects cell phenotype, determination, differentiation, and activation (Kajimura, 2015; Seale, 2015). Moreover, only EVs from TgTg increased the brown adipocyte activity, but the EVs from the WT did not. However, adding the concentrated EVs, from both WT and TgTg, to WT preadiopocytes showed increasing levels of *Ucp-1* and *Pgc-1α* expression in a dose-dependent manner (Figure 6H). This result is consistent with the actual measurement value of the number of EVs, which is 2- to 4-fold in TgTg compared to the WT (Figure 6D). During the secretome analysis, we did not see a major difference of between exosome content which contribute to brown adipocyte differentiation and activation (Table S1). Thus, it is the EV secretion capacity rather than EV content which may be the decisive factor in regulating brown adipocyte activity.

To date, BAT has been known to secrete many bioactive factors that activate BAT itself under thermogenic stimuli (Villarroya et al., 2013). The present study demonstrated that the EVs secreted by hTFAM-overexpressing brown adipocytes could permanently maintain the brown adipocyte phenotype as an active state even without stimuli, resulting in potent obesity resistance, locally, or at a distance. This study gives a good rationale for the stimulation of EV secretion from brown adipocytes for anti-obesity treatment. However, the direct effect between enhanced EV release and the beneficial effect on the metabolic phenotypes remain to be elucidated. As a next stage, we suggest further research into establishing the stable system to collect a large number of EVs to analyze the direct effects of EVs *in vivo*.

In this study, we discovered that the differentiation and activation of brown adipocyte are proceeded by EVs secreted from brown adipocyte in autocrine/paracrine and dose- dependent manners. In conclusion, our study demonstrated that the hTFAM overexpression in brown adipocytes activates adipocytes and increases mitochondrial functions via enhanced exosome secretion, which may contribute to obesity resistance.

### Limitations of the study

In the present study, we reveal that hTFAM TgTg has strong anti-obesity phenotype. Also we advocated that hTFAM overexpression in the brown preadipocytes accelerate mitochondrial function and EVs secretion, then facilitate self-preadipocyte differentiation as an anti-obesity mechanism. However, it has not yet been verified if the factor that influences on this phenotype is due to TFAM’s gain-of-function or loss-of-function. Therefore, it is required further verification with comparing the phenotype of this TgTg mice and another model, e.g. brown adipocyte specific conditional hTFAM knock-in mouse using ROSA 26 locus, to eliminate the possibility of off-target effects due to insertion of transgene construct, as a future study.

Another limitation to this study is that the number of brown adipocytes that could be used for transplantation was restricted. The weight loss effect was mild in the TgTg-t model compared to TgTg during HFD feeding. One reason for this difference is an insufficient number of transplanted cells because BAT in WT mice has at least 1×10^7^ brown adipocyte based on the size of brown cell pellet. Despite trying to transplant 1×10^7^ cells, the effect of BW loss was worse. Transplantation with a higher number of cells, therefore, may cause adverse effects possibly because of increased unknown artificial factors. We also found that it raised the immune reaction. Given that significant BW loss is obtained even with a transplantation of 1×10^6^ cells, hTFAM-overexpressing brown adipocytes partially play an important role against obesity. Moreover, we do not rule out the possibility that factors other than brown adipocytes also work against obesity in TgTg mice. Therefore, to eliminate these limitations, more *in vivo* studies are required to verify whether a BAT specific hTFAM-overexpressing model shows the same degree of obesity resistance as TgTg mice.

## STAR★Methods

### Key resources table

**Table:** 

REAGENT or RESOURCE	SOURCE	IDENTIFIER
**Antibodies**		

Human TFAM	Ikeuchi et al. (2005)	N/A
Mouse TFAM	Ikeuchi et al. (2005)	N/A
Rabbit monoclonal anti UCP-1	Abcam	Cat# ab209483; RRID:AB_2722676
Mouse monoclonal anti PGC-1α	Santa Cruz Biotechnology	Cat# sc-518038; RRID:AB_2895142
Rabbit polyclonal anti HSP90	Cell Signaling Technology	Cat# 4874; RRID:AB_2121214
Total OXPHOS Rodent WB Antibody Cocktail	Abcam	Cat# ab110413; RRID:AB_2629281
Rabbit monoclonal anti VDAC (D73D12)	Cell Signaling Technology	Cat# 4661; RRID:AB_10557420
Rabbit polyclonal anti HSP60 (D307)	Cell Signaling Technology	Cat# 4870; RRID:AB_2295614
Donkey polyclonal anti Rabbit IgG (H + L)	Jackson ImmunoResearch Labs	Cat# 711-036-152; RRID:AB_2340590
Goat polyclonal anti mouse IgG (H + L)	Jackson ImmunoResearch Labs	Cat# 115-036-062; RRID:AB_2307346
Histofine Simple Stain MAX PO(M)	Nichirei	Cat# 424131; RRID: AB_2313773

**Chemicals, peptides, and recombinant proteins**		

FBS (Exo-FBS^TM^ Exosome-depleted FBS)	SBI	Cat.#s Exo-FBS/HI 50A-1
Dispase II	gibco	REF 17105-041
Collagenase D	sigma	REF 11088858001
CaCl2	FUJIFILM Wako Pure Chemical Corporation	038-249985
Insulin	TAKARA	Cat# MK429-4
Triiodothyronine	TCI	Cas 6893-02-3
Indomethacin	FUJIFILM Wako Pure Chemical Corporation	PTF0267
Dexamethasone	TAKARA	Cat# MK429-2
Isobutylmethylxanthine [IBMX]	FUJIFILM Wako Pure Chemical Corporation	099-03411
Rosiglitazone	Almone labs	Cat# R-125
Glucose	FUJIFILM Wako Pure Chemical Corporation	049-31165
human biosynthetic insulin	Novo Nordisk	872492
Matrigel	Life Sciences	354234
Isoflurane inhalation solution	Pfizer	871119
Isogen	Nippon Gene	
Chloroform	FUJIFILM Wako Pure Chemical Corporation	038-02606
Isopropanol	FUJIFILM Wako Pure Chemical Corporation	166-04836
Gene-Packman Coprecipitant	Nacalai	12680-30
PrimeScript™ RT Reagent Kit	Takara Bio Inc.	Cat# RR036A
PerfeCTa PreAmp SuperMix	Quantabio	95146-040
GoTaq Real-Time PCR	Promega Corporation	A6001
RIPA buffer	Nacalai	08714-04
Methanol	FUJIFILM Wako Pure Chemical Corporation	137-01823
Bovine Serum Albumin	Nacalai	01281-26
Chemi-Lumi One Super	Nacalai	#02230
H_2_O_2_	FUJIFILM Wako Pure Chemical Corporation	081-04215
HCl	FUJIFILM Wako Pure Chemical Corporation	137-01823
Diaminobenzidine	Nichirei	415171
Trichloroacetic acid	FUJIFILM Wako Pure Chemical Corporation	208-08081
Trifluoroacetic acid	FUJIFILM Wako Pure Chemical Corporation	208-02746
Acetonitrile	FUJIFILM Wako Pure Chemical Corporation	019-21691
GW4869	Selleckchem.com	6823-69-4
Nexinhib20	CAYMAN CHEMICAL	331949-35-0

**Critical commercial assays**		

FGF-21 Mouse ELISA Kit	BioVendor	RD291108200R
Ultra-Sensitive Mouse Insulin ELISA Kit	Morinaga Institute of Biological Science, Inc.	M1104
Pierce^TM^ BCA Protein Assay Kit	Thermo Fisher Scientific	23227

**Experimental models: Organisms/strains**		

Mouse: human *TFAM* Tg	Ikeuchi et al. (2005)	N/A
C57BL/6 mice	Charles River	https://www.criver.com/products-services/find-model/jax-c57bl6j-mice?region=28

**Oligonucleotides**		

See Table S2 for primer sequences used for qRT-PCR analysis		

**Software and algorithms**		

NTA 3.2 software	Malvern Panalytical Ltd	https://www.malvernpanalytical.com/en/support/product-support/software/NanoSight-NTA-software-update-v3-2
ImageJ 1.47	Schneider et al., 2012	https://imagej.nih.gov/ij/
SPECTROstar Nano software Version 2.10	BMG LABTECH	https://www.bmglabtech.com/jp/microplate-reader-software/
ImageQuant™ LAS 4000 Version 1.2	GE Healthcare	https://imagequant-las-4000.software.informer.com/1.2/
StepOne™ Software Version 2.2.2	Thermo Fisher Scientific	https://www.thermofisher.com/jp/ja/home/technical-resources/software-downloads/StepOne-and-StepOnePlus-Real-Time-PCR-System.html
Proteome Discoverer software	ThermoScientific	N/A

**Other**		

Rodent diet CE-2	CLEA Japan, Inc.	
High Fat Diet (HFD32)	CLEA Japan, Inc.	
OXYMAX	Columbus Instruments	N/A
Falcon cell strainer (100μm)	Becton Dickinson	352360
UniWells™ Horizontal Co-Culture Plate	FUJIFILM Wako Chemicals U.S.A. Corporation	2501-02FW
Uniwells™ Filter 0.6μm	FUJIFILM Wako Chemicals U.S.A. Corporation	2525-06FW
Uniwells™ Filter 0.03μm	FUJIFILM Wako Chemicals U.S.A. Corporation	2525-003FW
StepOnePlus™ Real-Time PCR Systems	Thermo Fisher Scientific	https://www.fishersci.co.uk/gb/en/promotions/applied-biosystems-stepone-steponeplus-real-time-pcr-systems.html
SPECTROstar Nano	BMG LABTECH	https://www.bmglabtech.com/it/spectrostar-nano/
ImageQuant™ LAS 4000	GE Healthcare	GE Healthcare Systems | GE Healthcare (United States)
PVDF Transfer Membrane	Thermo Fisher Scientific	88518
Model BZ-9000	Keyence	N/A
MonoSpin TiO column	GL Sciences Inc.	1130007
LCMS-8060 Liquid Chromatography-Mass Spectrometer (LC/MS)	Shimadzu Corp.	N/A
transmission electron microscope (TEM) JEM-1400Plus	JEOL Ltd.	N/A
CCD camera EM-14830RUBY2	JEOL Ltd.	N/A
NanoSight NS300	Amesbury	N/A
ExoCounter	JVCKENWOOD Corporation	JEOL Ltd.
MonoSpin C18	GL Sciences	N/A
Easy-nLC1000 system	ThermoScientific	N/A
Acclaim PepMap100 trap column	ThermoScientific	N/A
Acclaim PepMap RSCL analytical column	ThermoScientific	N/A
Q-Exactive Orbitrap mass analyzer	ThermoScientific	N/A

### Resource availability

#### Lead contact

Further information and requests for resources and reagents should be directed to and will fulfilled by the lead contact, Dr. Masakazu Fujii (mafujii@med.kyushu-u.ac.jp).

#### Materials availability

This study did not generate new unique reagents.

### Experimental model and subject details

#### Mouse models

We bred and used human TFAM (hTFAM) transgenic mice originally generated in our laboratory by Ikeuchi et al. (2005). hTFAM cDNA was inserted into the unique EcoRI site between the CAG (modified chicken β-actin promoter with CMV-IE enhancer) promoter and 3′-flanking sequence of the rabbit β-globin gene of the pCAGGS expression vector and used to generate transgenic mice. The pronuclei of fertilized eggs from hyperovulated C57BL/6 mice were microinjected with this DNA construct. The presence of the TFAM transgenes was confirmed by polymerase chain reaction (PCR) before the experiments. Four independent founder lines were identified and mated to C57BL/6 wildtype (WT) mice to generate pure C57BL/6 genetic background hemizygous/homozygous hTFAM transgenic (Tg and TgTg, respectively) and non-transgenic WT offspring. The animals were allowed free access to water and a standard diet (SD, CE-2; 343 kcal/100 g, 12.6% energy as fat; CLEA Japan, Inc.). Eight-week-old male mice were fed a high-fat diet (HFD) (HFD 32 provided 507.6 Kcal/100 g [32.0% energy as fat; CLEA Japan, Inc.]) for 8 weeks. At the end of the experiment, the animals were sacrificed by isoflurane anesthesia inhalation after 16 h of fasting. Mice were housed 2–5 per cage at standard housing conditions (22°C, 12h light/dark cycle, relative humidity of 45–65 rH). All mice were kept under specific pathogen-free conditions in the animal facility at Kyushu University. All protocols were approved by the Committee on the Ethics of Animal Experiments at the Graduate School of Medical Sciences, Kyushu University.

#### Cell culture

##### Isolation of primary Brown preadipocytes

Primary brown preadipocytes were isolated as previously described (Aune et al., 2013). Briefly, adipose tissue was dissected from euthanized 6–8-week-old mice. Digestion medium (DMEM/F12 containing 10% FBS, P/S, 2.4 U/mL Dispase II, 0.2 W/V % Collagenase D and 10mM CaCl2: 1 mL/g tissue) was added to the tissue, and it was then minced into small pieces. A brownish pellet was obtained after digestion at 37°C with constant agitation at 200 rpm for 40 min. The cell suspension was filtered using a cell strainer (100 μm Falcon cell strainer) (Becton Dickinson, Lincoln Park, NJ, USA), and cells were plated on collagen I-coated dishes. Cells were washed twice with PBS, and fresh medium was added 1–2 h after plating the cells to remove red blood cells. All cells were grown at 37°C in a 5% CO_2_ humid atmosphere.

#### Transplantation

Primary brown preadipocytes (passage 4–6, 1 × 10^6^ cells) from WT or TgTg mice were induced to differentiation after 48 h of incubation in induction medium (DMEM/F12 [10% FBS and P/S], 5 μg/mL insulin, 1 nM Triiodothyronine [T3], 125 μM indomethacin, 2 μg/mL dexamethasone, 0.5 mM isobutylmethylxanthine [IBMX], 0.5 μM rosiglitazone). Those *in vitro* differentiated (also called “activated”) brown preadipocytes were suspended in Matrigel (Corning, NY, USA). Eight-week-old male mice were sub-dermally injected with the cell suspension on the dorsal body surface above the BAT under anesthesia. After transplantation, mice were fed an HFD for 8 weeks. At the end of the experiment, the animals were sacrificed by the inhalation of isoflurane after 16 h of fasting.

### Method details

#### Respiratory metabolism

The metabolic rate was measured by indirect calorimetric analysis in WT, Tg, and TgTg mice using an open-circuit calorimeter (Oxymax; Columbus Instruments, Columbus, OH, USA). Sixteen-week-old mice, after 8 weeks on a HFD, were housed in individual chambers. The temperature was maintained at 22 °C, with an airflow of 0.5 L/min. The animals were allowed free access to water and a standard diet. Mice were acclimatized to the chambers for 24 h before monitoring. They were then monitored for 72 h VO_2_ and VCO_2_ were measured every 10 min using an electrochemical O_2_ analyzer and CO_2_ sensor (Oxymax), and the RER was calculated as VCO_2_/VO_2_ (volume of CO_2_ produced per volume of O_2_ consumed [mL/Kg/h]).

#### RNA extraction and quantitative RT-PCR

Total RNA was extracted from frozen adipose tissue and adipocyte samples using the Isogen reagent (Nippon Gene, Tokyo, Japan), according to the manufacturer’s instructions. Extracted RNA (1 μg) was converted into single-stranded cDNA using a PrimeScript™ RT Reagent Kit (Takara Bio Inc., Tokyo, Japan). mRNA levels were quantified by quantitative RT-PCR using a GoTaq Real-Time PCR (Promega Corporation, Madison, WI, USA) and the StepOnePlus™ Real-Time PCR Systems (Thermo Fisher Scientific). The primers used for each target gene are listed in Table S2.

#### Western blot analysis

For total protein extract and western blot analysis, hTFAM, mouse TFAM, UCP-1, PGC-1α, OXPHOS, inguinal white adipose tissue (i-WAT), epididymal white adipose tissue (e-WAT), and BAT were homogenized in RIPA buffer (Nakarai, Kyoto, Japan), and centrifuged for 10minat 13,000 rpm. Protein concentrations were determined using a Pierce^TM^ BCA Protein Assay Kit (Thermo Fisher Scientific); 20 μg protein/lane was separated discontinuously on sodium dodecyl sulfate-polyacrylamide gels (10%) and transferred to PVDF 0.45μm membranes (Millipore, USA). After blocking nonspecific binding sites, membranes were incubated overnight at 4 °C with primary antibodies, followed by HRP-conjugated secondary antibodies. The primary and secondary antibodies are listed in the key resources table. Chemi-Lumi One Super (Nakarai, Kyoto, Japan) was used for the detection.

#### Measurement of blood glucose, intraperitoneal glucose tolerance test (IPGTT), and intraperitoneal insulin tolerance test (IPITT)

Blood samples were obtained from the tail vein of the mice. Plasma glucose and insulin concentrations were determined using the glucose oxidase method and an enzyme-linked immunosorbent assay (ELISA; Morinaga Institute of Biological Science, Yokohama, Japan), respectively. After 16 h of fasting, glucose tolerance was assessed by an IPGTT, and a glucose bolus (5 mg/kg BW) was injected intraperitoneally. For the IPITT, mice were intraperitoneally injected with human biosynthetic insulin (0.5 U/kg BW) (Novo Nordisk, NJ, USA).

#### Morphometric study

BAT, iWAT, and eWAT were stained with hematoxylin and eosin (HE). Mouse adipose tissues were obtained after 8 weeks of a normal chow diet (NCD) or HFD conditions. To calculate the adipocyte area, the sections were coded and analyzed by a blinded observer. In each animal from the four experimental groups, 2,000 or more adipocytes in 24 randomly selected fields at 200-fold magnification using fluorescent microscopy (Model BZ-9000, Keyence, Osaka, Japan) were examined and averaged for morphometric analysis. Immunostaining for hTFAM in BAT was performed as previously described (Ikeuchi et al., 2005). Briefly, the BAT were fixed in 10% formaldehyde and embedded in paraffin. Paraffin sections were cut at 3 μm and deparaffinized. After inactivation of endogenous peroxidase with 10% H_2_O_2_ in methanol for 20 min at room temperature, the sections were preincubated for 30 min with 1% bovine serum albumin in phosphate-buffered saline (PBS). The samples were then incubated with anti-hTFAM affinity purified antibody (4 μg/mL) overnight at 4°C, washed in PBS, and probed with anti-mouse IgG antibody labeled with peroxidase (Histofine Simple Stain MAX PO(M)) for 30 min at room temperature. The peroxidase was then visualized with diaminobenzidine. The primary antibody was replaced with mouse IgG as a negative control.

BAT, including the Matrigel part, was briefly fixed in 10% formaldehyde and embedded in paraffin. Paraffin sections were cut to 3 μm thickness and deparaffinized. After inactivation of endogenous peroxidase with 10% H_2_O_2_ in methanol for 20 min at room temperature, the sections were preincubated for 30 min with 1% bovine serum albumin in phosphate-buffered saline (PBS). The samples were then incubated with anti-human TFAM mouse monoclonal antibody (4 μg/mL) overnight at 4°C, washed in PBS, and probed with anti-mouse IgG antibody labeled with peroxidase (Histofine Simple Stain MAX PO(M), Nichirei Biosciences Inc., Tokyo, Japan) for 30 min at room temperature. The peroxidase sites were visualized with diaminobenzidine (Nichirei Biosciences Inc., Tokyo, Japan).

#### Urine analysis

A 24-h urine sample was collected, and an acidic urine analysis was conducted with 6N HCl using metabolic cages. Urinary dopamine, noradrenalin, and adrenalin were analyzed by high-performance liquid chromatography (HPLC). The urine was then mixed and centrifuged at 7500× g for 5 min before being stored at −80°C for analysis.

#### Nucleotide purification

Nucleotides were extracted from WT and TgTg preadipocytes using 2% trichloroacetic acid (TCA). The sample solution was incubated on ice for 3 min and then centrifuged at maximum rpm for 5minat 4 °C. Then, 0.1% trifluoroacetic acid (TFA) in 50% acetonitrile (ACN) was added to the supernatants, and the mixture was transferred to a MonoSpin TiOspin column (GL Sciences Inc., Tokyo, Japan) and centrifuged at 5000×g for 2 min. Next, 0.1% TFA in 80% ACN was added, the solution was centrifuged at 5000×g for 2 min, 2.5% 50 μL of aqueous ammonia solution was added, and it was centrifuged at 1000×g for 1 min. The purified sample was collected with an equivalent volume of ACN and then injected into a liquid chromatography-mass spectrometer (LC/MS).

#### Medium transplantation study

WT cells were cultured until confluent in a 6-well plate covered with collagen I. The cells were then washed with PBS and the supernatants from TgTg cells culture were added, which was collected 7 days after confluent condition. After transplantation, the differentiated cells, which could be detected via the apparent lipid droplets, were counted until day 7.

#### Co-culture study

We evaluated the contribution of EVs to adipocyte differentiation using a horizontally connected co-culture system that allowed for the simultaneous observation of both culture vessels. The same numbers of WT and TgTg cells were seeded into the UniWells™ Horizontal Co-Culture Plate (FUJIFILM Wako Chemicals USA. Corporation), separated by a 0.03 or 0.6 μm pore size filter. Both cells were collected 10 d after incubation at 37°C, and mRNA was extracted. We also performed this with the co-culture cells, 4 days after the establishment of the confluent condition, and with the matured 3T3-L1 cells, 7 days from the beginning of differentiation. 3T3-L1 cells were seeded and differentiated on the UniWells™ Horizontal Co-Culture Plate before being connected to the TgTg cell plate.

#### Electron microscopy

The samples were fixed with 2% paraformaldehyde (PFA) and 2% glutaraldehyde (GA), post-fixed with 2% osmium tetroxide and embedded in resin. The polymerized resins were ultra-thin sectioned at 70 nm using an ultramicrotome. They were stained with 2% uranyl acetate and further stained with a lead stain solution. The grids were observed using a transmission electron microscope (TEM; JEM-1400Plus; JEOL Ltd., Tokyo, Japan) at an acceleration voltage of 100 kV. Digital images were captured using a CCD camera (EM-14830RUBY2; JEOL Ltd., Tokyo, Japan).

#### Exosome isolation

Cells were cultured until confluent in 100 mm dishes, then washed with PBS and added to the medium supplemented with EV depleted FBS. Vesicle depletion in FBS was performed via ultracentrifugation at 100,000 × g at 4 °C for 16 hours. The cell number in both WT and TgTg were approximately 1×10^6^ per 100mm dish. Cell culture conditioned media were harvested 48 h after changing medium and centrifuged at 12,000 rpm for 1 h at 4°C to remove contaminating apoptotic bodies and cell debris. The supernatants were transferred to a clean tube and centrifuged again at 34,500 rpm for 2 h at 4°C to pellet the exosomes. The supernatants were carefully removed, and exosome-containing pellets were resuspended in 200 μL of PBS and pooled.

#### Nanoparticle tracking analysis (NTA)

NTA measurements were performed using a NanoSight NS300 Instrument (NanoSight, Amesbury, UK). After resuspending exosome pellets in 200 μL of PBS, samples were diluted 1000-fold with PBS before measurement. Particles in the laser beam underwent Brownian motion, and a video of the particle movements was recorded. NTA 3.2 software was used to analyze the video and to determine particle size distribution. Twenty-five frames per second were recorded for each sample at appropriate dilutions at the “frames processed” settings of 1,500. The detection threshold was set at “5 Multi,” and at least 1,000 tracks were analyzed for each video.

#### Exosome count

The exosomes were counted as described previously (Kabe et al., 2018). The optical disc was attached to a removal plate containing 16 wells for sample injection. Each well was coated with 5 mg/L anti-CD9 or anti-CD63 antibody in a carbonate-bicarbonate buffer (pH9.6) overnight at 4°C. After removing the carbonate-bicarbonate buffer and washing with 0.05% Tween 20 in PBS (PBS-T), blocking solution (0.1% casein in PBS-T) was added and incubated for 30 min at 37°C. Next, 50 μL of the sample solution was added to each well and incubated for 2 h at 37°C followed by washing with PBS-T. Then, approximately 1 μg of anti-CD9 or anti-CD63 antibody-attached beads in blocking solution was added to each well and incubated for 90 min at 37°C. Each well was washed with PBS-T, followed by PBS-T and deionized water. The discs were dried in a thermostatic oven at 37°C for 10 min for measurements using an ExoCounter (JVCKENWOOD Corporation, Yokosuka, Japan).

#### Exosome addition

Purified exosome, from WT or TgTg brown preadipocytes was concentrated at each magnification (0.5, 1.0, and 2.0×), and added to WT brown preadipocytes, 4 days after the establishment of the confluent condition. Each cell was harvested 7 days later and evaluated for brown adipocyte differentiation/activation and related gene expressions.

#### Trypsin digestion and sample preparation for mass spectrometry (MS) analysis

Proteins derived from EVs were lysed with lysis buffer containing 8M urea and 500mM Tris-HCl (pH8.0) and reduced with DTT (final 5 mg/mL) at 37°C for 30 min, subjected to carbamidomethylation of cysteine using iodoacetamide (final 8 mg/mL) at 37°C for 30 min, and then diluted with 4 vol of 50mM ammonium bicarbonate. The protein solution was subjected with trypsin digestion using MonoSpin Trypsin and the digests were purified using MonoSpin C18 (GL Sciences), according to the manufacturer’s instruction, respectively. The methanol eluate was evaporated and then dissolved with 0.1% formic acid containing 2% acetonitrile.

#### LC-MS/MS analysis

LC-MS/MS analysis was performed as previously described (Matsushima et al., 2021). Briefly, approximately 1 g of peptide was separated with the Easy-nLC1000 system (ThermoScientific) using Acclaim PepMap100 trap column (20 × 0.075mm, 3um, Thermo Scientific) and the Acclaim PepMap RSLC analytical column (150 × 0.05mm, 2um, Thermo Scientific) and analyzed on Q-Exactive Orbitrap mass analyzer (Thermo Scientific). Data analysis was performed using Proteome Discoverer software (Thermo Scientific) for protein identification through SequestHT algorithm against human protein Uniprot database.

#### Exosome inhibitor

GW4869 and nexinhib20 were used to investigate the role of exosomes in inducing differentiation and activation in TgTg brown preadipocytes. Brown preadipocytes were briefly seeded with exosome-free medium (complete medium containing exosome-free FCS) and cultured for 2 d (confluent). For the GW4869 treatment, the culture medium was replaced with an exosome-free medium containing 20 μM GW4869 and cultured for 3 d. The medium was then replaced with an exosome-free medium and cultured for 4 d. For the nexinhib20 treatment, the culture medium was replaced with an exosome-free medium containing 2 μM nexinhib20 and cultured for 2 d. The medium was then replaced with an exosome-free medium and cultured for 5 d.

### Quantification and statistical analysis

The data displayed represent at least three independent experiments, unless otherwise specified. Data are expressed as the mean ± SE. Differences between groups were analyzed using a two-tailed Student’s *t* test with 95% confidence interval. ANOVA with 95% confidence interval was used to compare three or more comparable groups. Statistical comparisons were completed using the GraphPad Prism 8 software.

## Data Availability

• All data reported in this paper will be shared by the lead contact on request. • This paper does not report original code. • Any additional information required to reanalyze the data reported in this paper is available from the lead contact on request.

## Supplemental information

Document S1. Figures S1–S8

Table S1. Secretome analysis in the TgTg and WT adipocyte culture medium, related to Figure 6

Table S2. RT-qPCR primers used in this study, related to STAR Methods, Figure 3–6 and Figures S3–S5
