# Supplementary material for: TFAM expression in brown adipocytes confers obesity resistance by secreting extracellular vesicles that promote self-activation
Source: iScience. 2022 Aug 10;25(9):104889. doi: 10.1016/j.isci.2022.104889 (PMC9421388; doi:10.1016/j.isci.2022.104889)
Supplement: Document S1. Figures S1–S8 [file mmc1.pdf]

iScience, Volume *25*

Supplemental information

TFAM expression in brown adipocytes confers obesity resistance by secreting extracellular vesicles that promote self-activation

Masakazu Fujii, Daiki Setoyama, Kazuhito Gotoh, Yushi Dozono, Mikako Yagi, Masataka Ikeda, Tomomi Ide, Takeshi Uchiumi, and Dongchon Kang

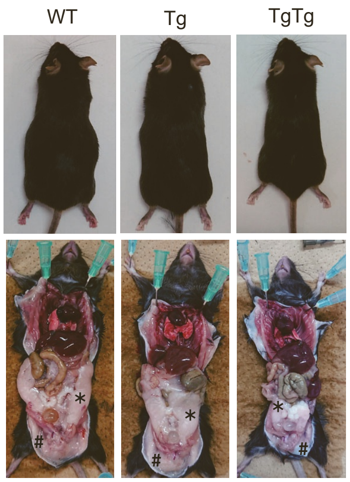

Figure S1 (related to Fig.1): Metabolic characterization of WT, Tg, and TgTg mice under the NCD condition TgTg mice had extremely low i (#)- and e (*)-WAT accumulation on the NCD compared to WT mice.

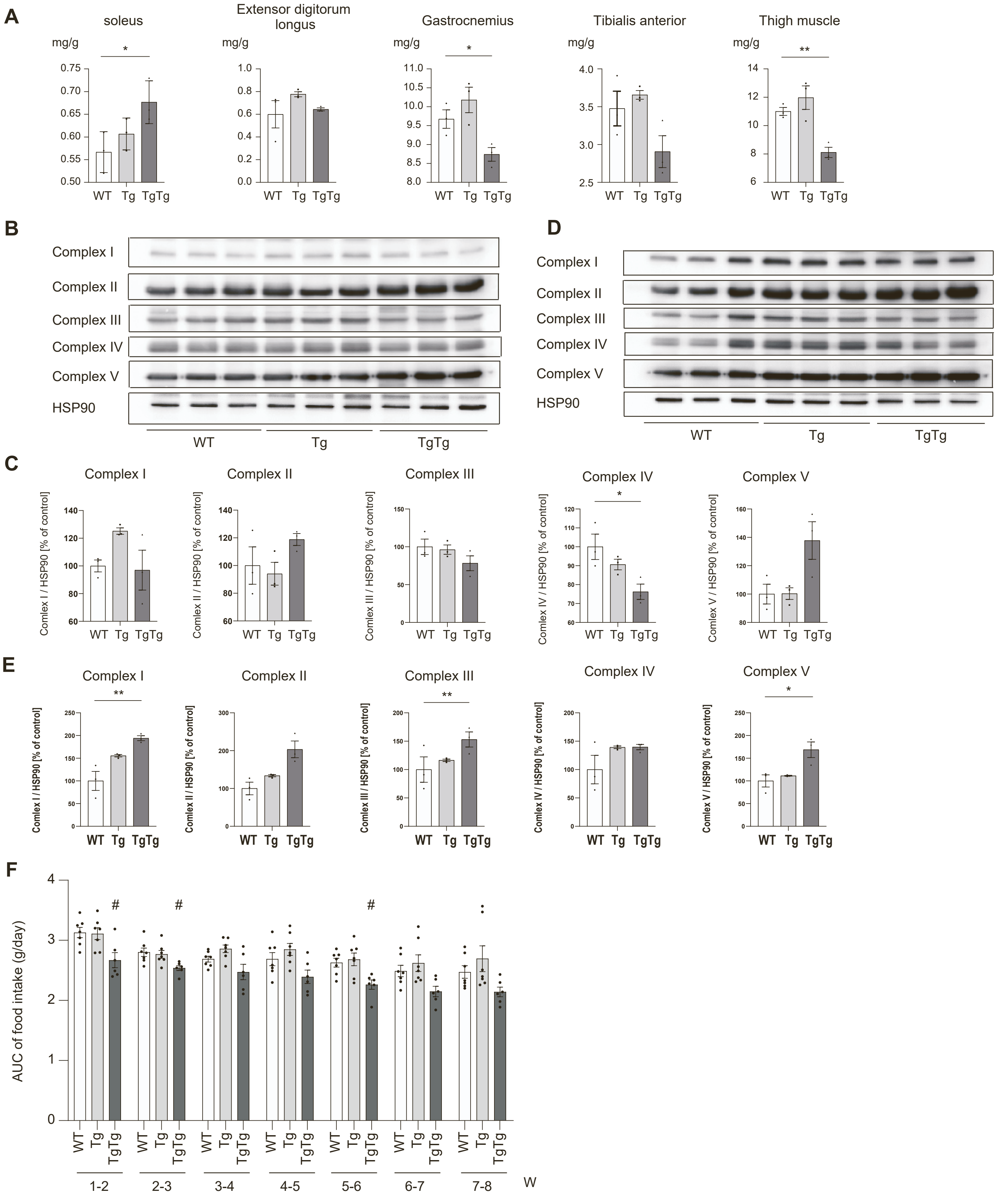

Figure S2 (related to Fig.2): Metabolic characterization of WT, Tg, and TgTg mice under the HFD condition
(A) Weight per body of the lower limb muscles. Mitochondrial OXPHOS protein expression in the (B, C) soleus and (D, E) extensor digitorum longus muscle (n = 3 / group). *P< 0.05, **P < 0.01 vs WT. (F) Changes in food intake analyzed by AUC (n = 5–10 / group). #P<0.05 vs WT. One-way ANOVA with Turkey' s multiple comparisons test in (A), (C), (E) and (F). Bar graphs represent mean ± SE.

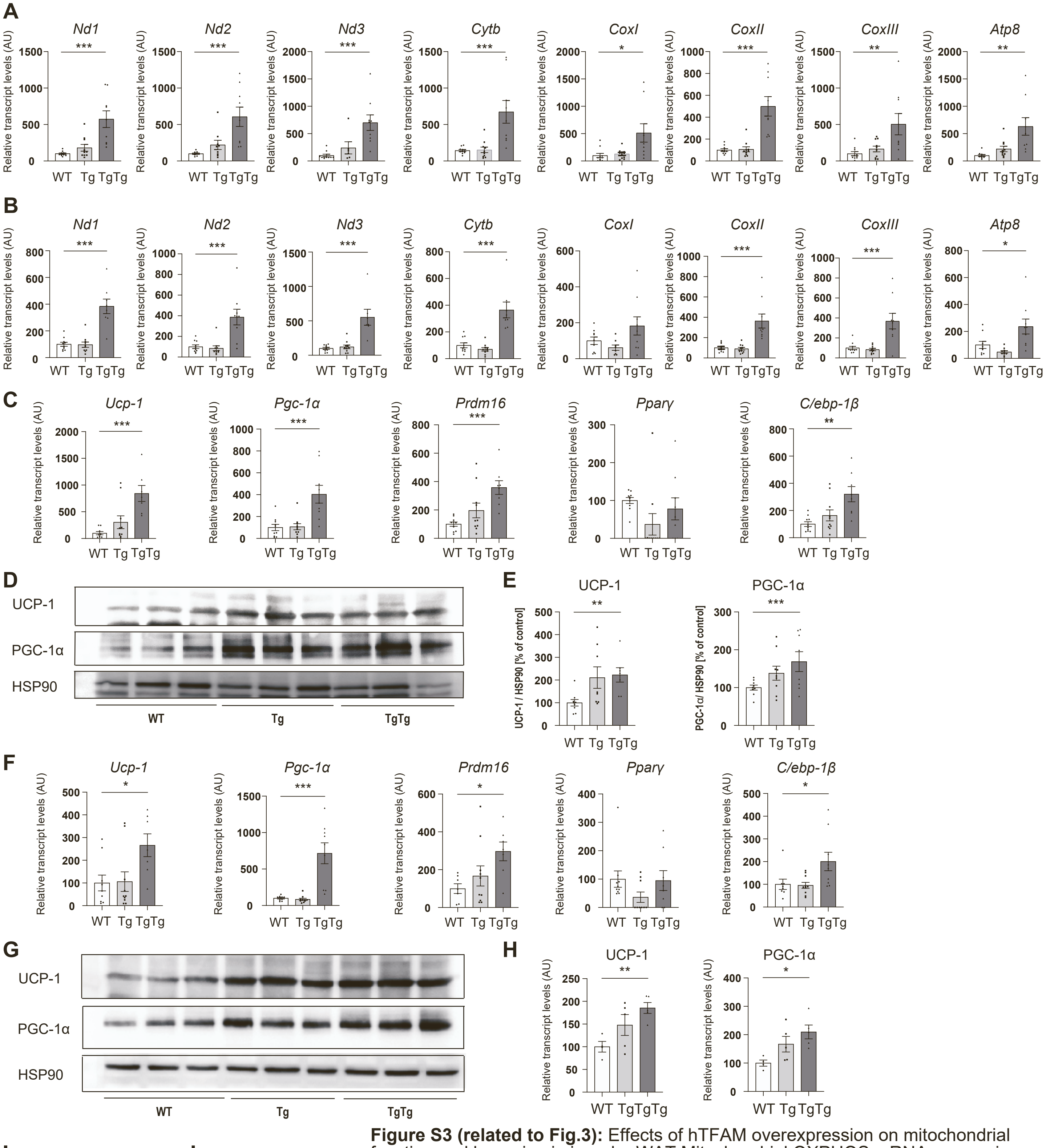

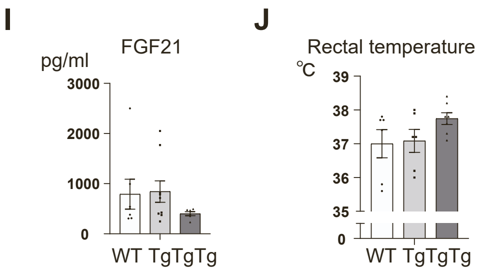

Figure S3 (related to Fig.3): Effects of hTFAM overexpression on mitochondrial function and browning in i- and e-WAT Mitochondrial OXPHOS mRNA expression in (A) i-WAT and (B) e-WAT. Total RNA extracted from HFD-fed WT, Tg,and TgTg mice. All mRNA levels were measured by RT-PCR. Levels of mRNA were normalized to the levels of *Hprt*, and the results are expressed as the mean percentage of the level in control WT mice ± SE (n = 8–10 / group). Thermogenic and browning-regulating genes in (C) i-WAT and (F) e-WAT were measured by RT-PCR (n = 7–10 / group). UCP-1 and PGC-1α protein levels were evaluated using western blot analysis. Pictures represent western blots for UCP-1 and PGC-1α in (D) i-WAT and (G) e-WAT. Quantifications were normalized to the level of HSP90. The results are expressed as the mean percentage of the level in the WT ± SE in (E) i-WAT (n = 7–9 / group) and (H) e-WAT (n = 4–5 / group). (I) Circulating FgF21 levels. (J) The deep body temperature (rectal temperature). *P< 0.05, **P < 0.01, ***P < 0.001 vs WT. One-way ANOVA with Turkey’ s multiple comparisons test in (A) - (C), (E), (F) and (H) – (J).

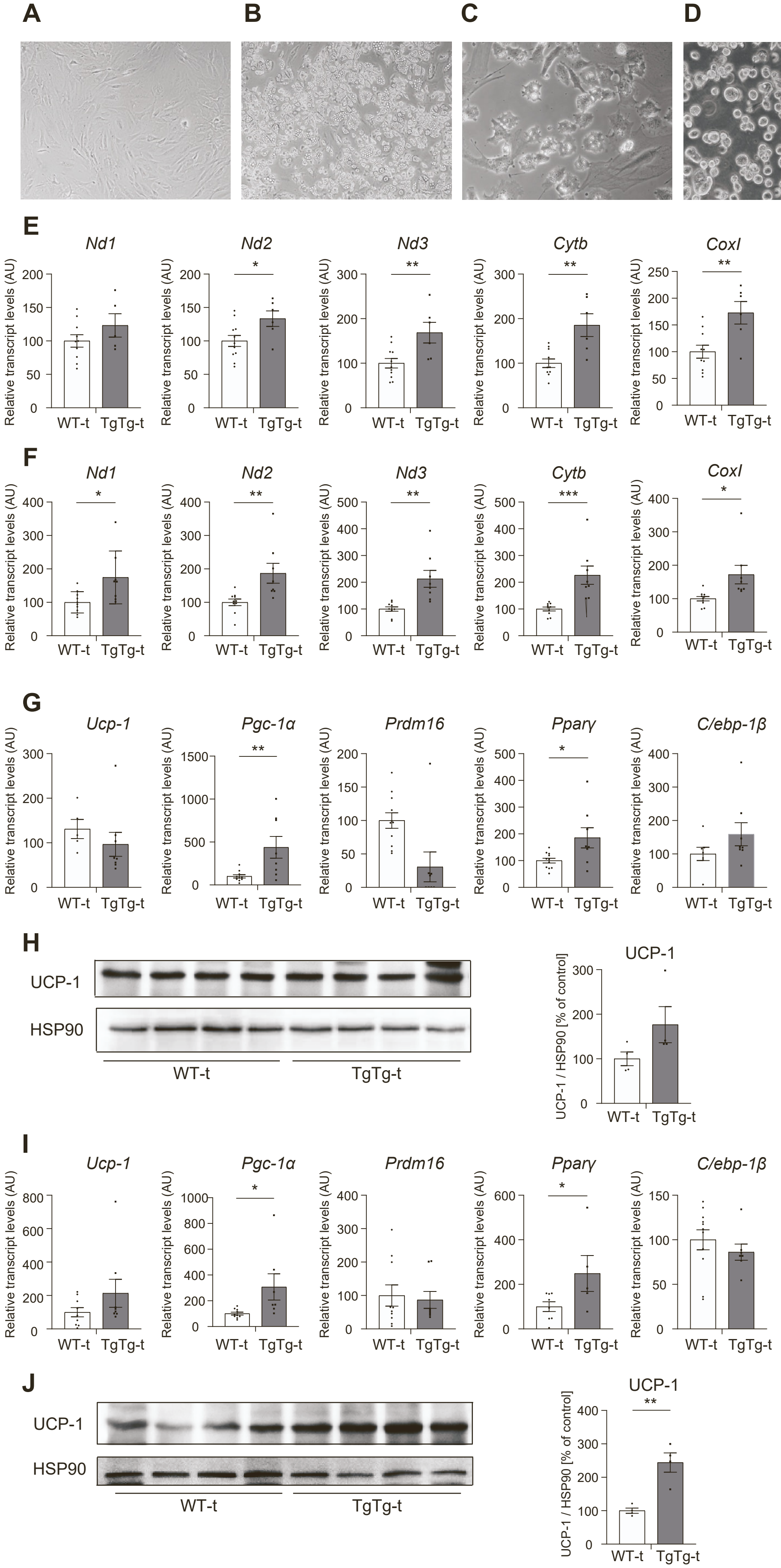

Figure S4 (related to Fig.4): Effects of hTFAM-overexpressing brown adipocyte transplantation on WAT browning in HFD-fed mice (A) Pictures represent the non-activated brown preadipocytes (X40); (B) Activated brown adipocytes (X40); (C) (X 200); and (D) floating (trypsinized) activated adipocytes (X200). Mitochondrial OXPHOS mRNA expression in (E) i-WAT and (F) e-WAT. Total RNA extracted from the HFD-fed WT-t and TgTg-t. All mRNA levels were measured by RT-PCR. The mRNA levels were normalized to the levels of *Hprt*, and the results are expressed as the mean percentage of the control WT-t ± SE (n = 6–10 / group). The mRNA levels of thermogenic and browning markers in (G) i-WAT and (I) e-WAT were assessed by RT-PCR (n = 6–10 / group). UCP-1 protein levels were evaluated using western blot analysis. Pictures represent western blots for UCP-1 in (H) i-WAT and (J) e-WAT. Quantifications were normalized to the level of HSP90. The results are expressed as the mean percentage of the level in the WT ± SE in (H) i-WAT (n = 4 / group) and (J) e-WAT (n = 4 / group). *P< 0.05, **P < 0.01, ***P < 0.001 vs WT - t. Two-tailed unpaired Student' s t test in (E) - (J).
Bar graphs represent mean ± SEM.

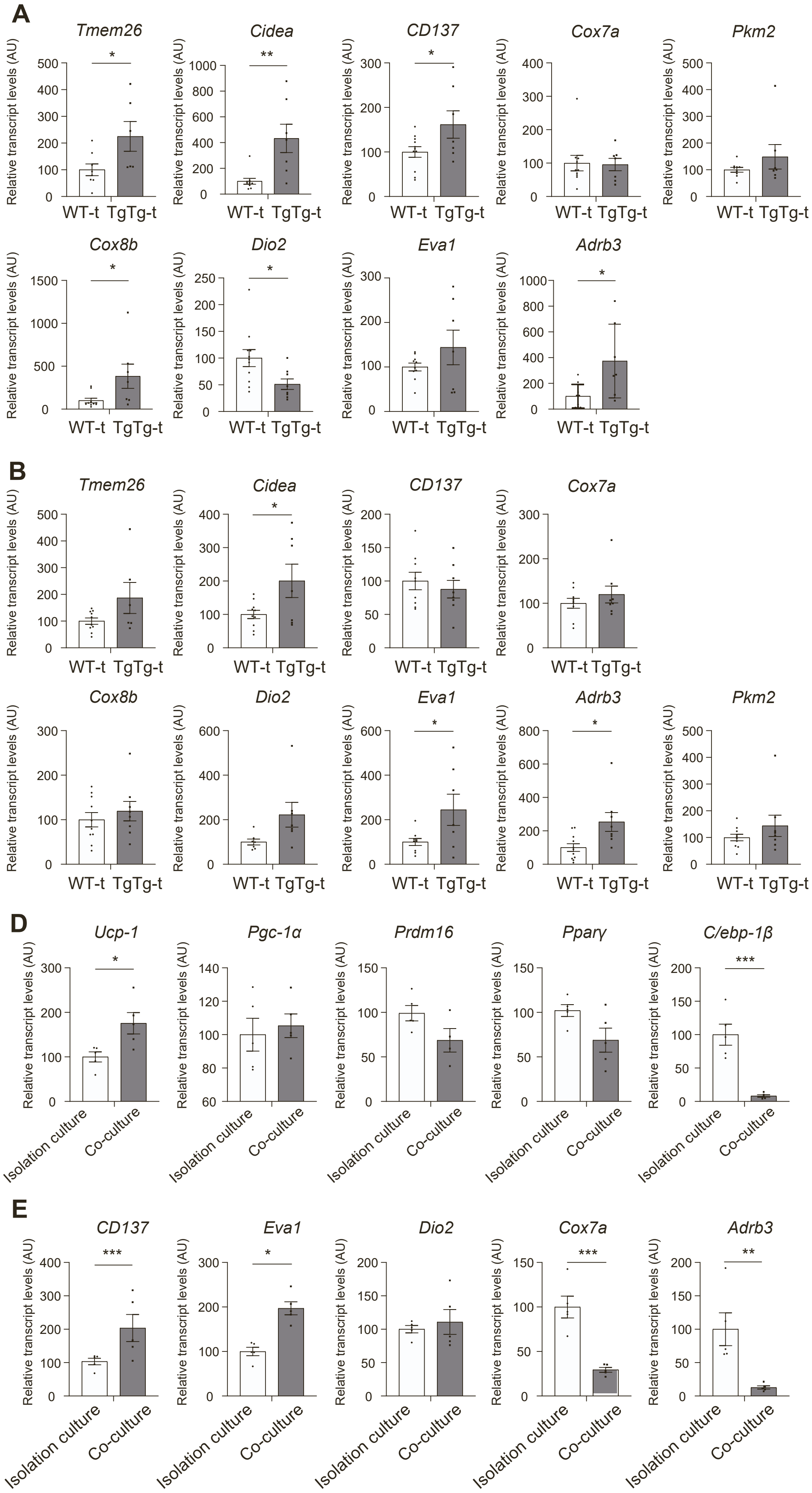

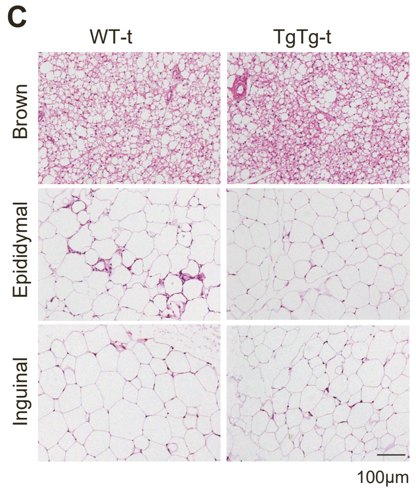

Figure S5 (related to Fig.4): Effects of hTFAM-overexpressing brown adipocyte transplantation on WAT browning in HFD-fed mice. The mRNA levels of beige adipocyte makers in (A) i-WAT and (B) e-WAT were assessed by RT-PCR (n = 6–10 / group). Total RNA extracted from the HFD-fed WT-t and TgTg-t. All mRNA levels were measured by RT-PCR. The mRNA levels were normalized to the levels of *Hprt*, and the results are expressed as the mean percentage of the control WT-t ± SE (n = 6–10 / group). *P< 0.05, **P < 0.01, ***P < 0.001 vs WT - t. Two-tailed unpaired Student' s t test in (A) - (B). Bar graphs represent mean ± SEM. (C) Microscopic e- and i-WAT and BAT sections stained with hematoxylin and eosin. Scale bars represent 100 μm. The mRNA levels of (D) thermogenic / browning markers and (E) beige adipocyte makers in isolation and co-culture matured 3T3-L1 adipocyte were assessed by RT-PCR (n = 4 – 5 / group). *P< 0.05, **P < 0.01, ***P < 0.001 vs Isolation culture. Two-tailed unpaired Student' s t test i n (D) (E).

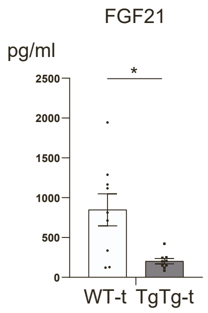

Figure S6 (related to Fig.4): Circulating FGF21 levels in WT–t and TgTg–t.
*P< 0.05 vs WT-t. Two-tailed unpaired Student’ s t test.

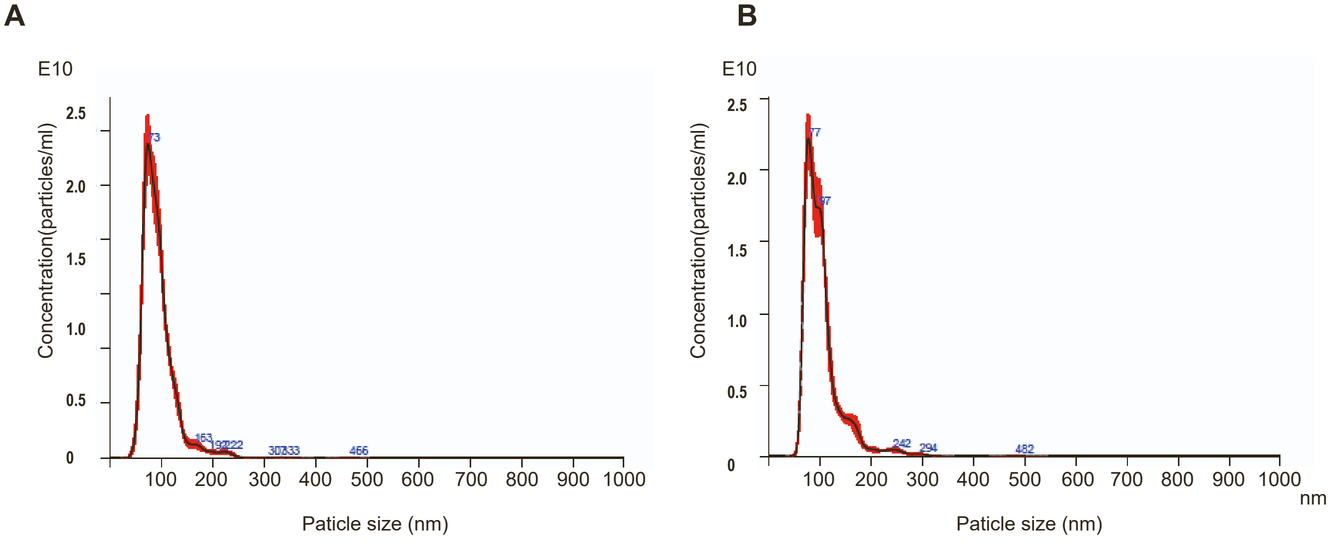

Figure S7 (related to Fig.6): Analysis of particle diameters
Particle diameter purified from (A) WT and (B) TgTg cell culture supernatants as analyzed by NanoSight.

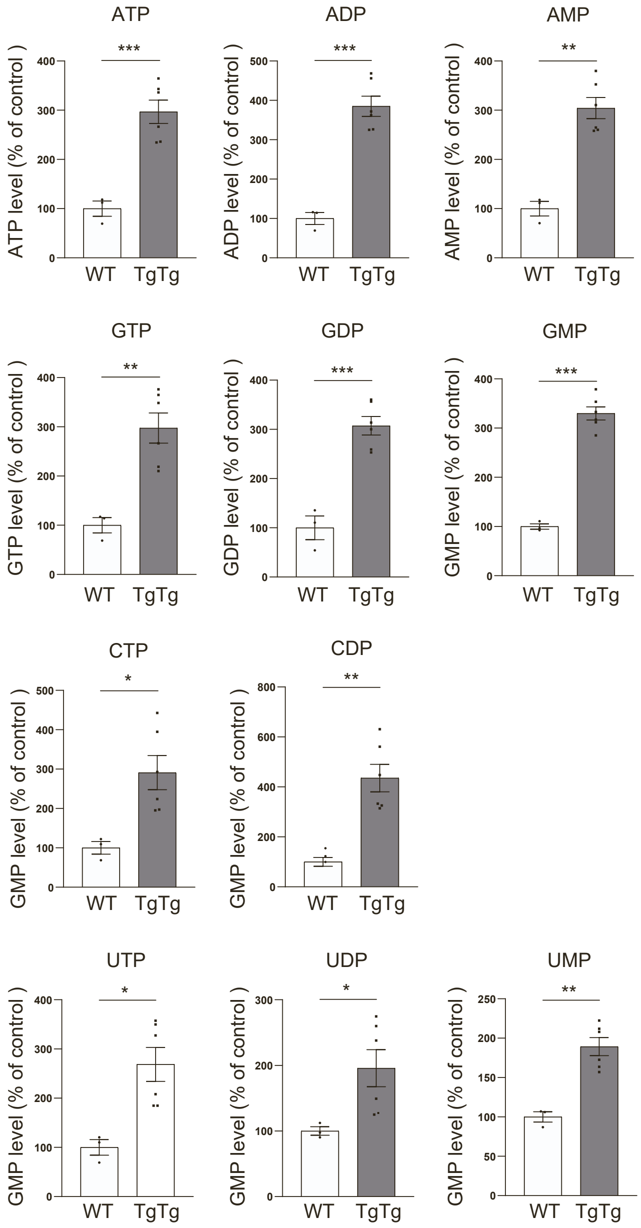

Figure S8 (related to Fig.6): Overproduction of nucleotides in TFAM TgTg brown adipocytes
Nucleotide levels in WT and TgTg brown preadipocytes measured by LC/MS (n = 3–6 / group).
*P< 0.05, **P< 0.01, ***P< 0.01 vs WT. Two-tailed unpaired Student’ s t test.
Bar graphs represent mean ± SEM.
